# Associations between body fat anthropometric indices and mortality among individuals with metabolic syndrome

**DOI:** 10.1186/s12944-024-02272-0

**Published:** 2024-09-27

**Authors:** Jianyou Shi, Zhiyuan Chen, Yuanfeng Zhang

**Affiliations:** 1 Department of Clinical Laboratory, The Third Affiliated Hospital of Shanghai University, Wenzhou, 325000 P. R. China; 2 Department of Clinical Laboratory, Wenzhou People’s Hospital, Wenzhou, 325000 P. R. China; 3 Department of Clinical Laboratory, Wenzhou Third Clinical Institute Affiliated Wenzhou Medical University, Wenzhou People’s Hospital, Wenzhou, 325000 P. R. China; 4 Department of Pediatrics, The Third Affiliated Hospital of Shanghai University, Wenzhou, 325000 Zhejiang P. R. China; 5 Department of Pediatrics, Wenzhou People’s Hospital, Wenzhou, 325000 P. R. China; 6 Department of Pediatrics, Wenzhou Third Clinical Institute Affiliated to Wenzhou Medical University, Wenzhou People’s Hospital, Wenzhou, 325000 P. R. China; 7 Department of Urology, Shantou Central Hospital, Shantou, 515000 P. R. China

**Keywords:** NHANES, A body shape index, Metabolic syndrome, Mortality

## Abstract

**Background:**

The distribution of body fat and metabolic health may contribute to the onset of metabolic syndrome (MetS), but the associations between body fat anthropometric indices (AIs) and mortality in individuals with MetS remain unclear.

**Methods:**

Participants aged 18 years or older with MetS were recruited from the NHANES 1999–2018. The body fat anthropometric indices included the a body shape index (ABSI), body roundness index (BRI), cardiometabolic index (CMI), visceral adiposity index (VAI), waist triglyceride index (WTI), lipid accumulation product (LAP), atherogenic index of plasma (AIP), and triglyceride‒glucose (TyG) index. MetS was defined according to the National Cholesterol Education Program Adult Treatment Panel III (NCEP ATPIII) criteria. Mortality data were obtained from the National Death Index through December 31, 2019.

**Results:**

Data were collected from 8,379 individuals with MetS, with a median follow-up of 8.5 years, of whom 1,698 died from all causes and 568 from the CCD. The random survival forest (RSF) analysis indicated that the ABSI had the strongest predictive power for both all-cause mortality and CCD mortality among the eight body fat AIs. After adjusting for multiple variables, the ABSI was found to be linearly and positively associated with all-cause and CCD mortality in individuals with MetS. Participants in the highest quartile of ABSI had an increased risk of all-cause (HR = 1.773 [1.419–2.215]) and CCD (HR = 1.735 [1.267–2.375]) mortality compared with those in the lowest quartile. Furthermore, the ABSI predicted areas under the curve (AUCs) of 0.735, 0.723, 0.718, and 0.725 for all-cause mortality at 3, 5, 10, and 15 years, respectively, and 0.774, 0.758, 0.725, and 0.715 for CCD mortality, respectively.

**Conclusion:**

Among eight body fat AIs, the ABSI exhibited the strongest predictive power for mortality in individuals with MetS. Higher ABSI values significantly increased all-cause mortality and CCD mortality in participants with MetS.

**Supplementary Information:**

The online version contains supplementary material available at 10.1186/s12944-024-02272-0.

## Introduction

Metabolic syndrome (MetS) is a cluster of conditions primarily characterized by insulin resistance, central obesity, hypertension, dyslipidemia, and other metabolic abnormalities [1]. The etiology of MetS is multifaceted and involves genetic, environmental, and lifestyle factors. Its pathogenesis is attributed mainly to insulin resistance, inflammatory responses, and oxidative stress [2]. The global prevalence of MetS has increased significantly, affecting approximately one-third of the world’s population [3]. Obesity, a hallmark of MetS, is widely recognized as a major risk factor for cardiovascular disease (CVD), diabetes mellitus, and certain cancers [4]. However, the concern in MetS extends beyond obesity to encompass the various metabolic abnormalities that accompany it [5]. These abnormalities include hypertension, hyperinsulinemia, insulin resistance, and dyslipidemia [6]. The interaction of these factors significantly elevates the risk of CVD and all-cause mortality in individuals with MetS [7].

Owing to the multiple metabolic abnormalities present in patients with MetS, relying solely on traditional indicators such as body mass index (BMI) may be insufficient for assessing their prognosis [8]. Recently, several studies have focused on other obesity-related AIs to better assess the link between obesity and chronic diseases. The a body shape index (ABSI) is an indicator that incorporates height, weight, and waist circumference (WC) to provide a more comprehensive assessment of an individual’s body shape and obesity level [9]. The body roundness index (BRI) has been shown to be a good predictor of metabolism-related diseases [10]. The cardiometabolic index (CMI), a novel index developed on the basis of triglyceride/high-density lipoprotein cholesterol (TG/HDL-C) and waist-to-height ratio (WHtR) values, is closely associated with obesity-related metabolic disorders [11]. The visceral adiposity index (VAI) and waist triglyceride index (WTI) reflect the accumulation of visceral fat and waist fat, respectively [12, 13]. The lipid accumulation product (LAP) combines measurements of WC and TG levels to reflect lipid accumulation in the body [14]. The atherogenic index of plasma (AIP), a lipid ratio, accurately reflects the balance between protective and atherogenic lipoproteins [15]. The triglyceride‒glucose (TyG) index combines TG and glucose levels to assess the degree of insulin resistance and abnormal glucose metabolism [16]. These metrics provide additional information about body fat distribution and types of obesity that may be more accurate than traditional metrics in predicting the risk of death associated with MetS.

Several body fat AIs are strongly associated with the development of MetS. Sugiura et al. demonstrated that ABSI can be utilized to identify individuals with MetS among middle-aged adults [17]. Rico-Martín et al. reported that the BRI significantly outperforms BMI, the waist-to-hip ratio (WHR), and the body adiposity index (BAI) in predicting MetS [18]. Additionally, a study by MS et al. revealed that the AIP was significantly elevated in patients with MetS, with the highest area under the curve for identifying MetS [15]. Although these studies explored the relationships between certain body fat AIs and MetS, the associations between these indices and mortality in patients with MetS have not been adequately investigated. Therefore, this study hypothesizes that specific body fat indices (ABSI, BRI, CMI, VAI, WTI, LAP, AIP, and TyG) are significant predictors of mortality in individuals with MetS. To evaluate this, this study analyzed the associations between these body fat indices and mortality and identified the most effective predictors. By doing so, this study seeks to deepen the understanding of mortality risks associated with obesity in MetS patients, providing valuable insights for improved management and prognosis of this syndrome.

## Materials and methods

### Study population

The National Health and Nutrition Examination Survey (NHANES), conducted biennially by the National Center for Health Statistics (NCHS) [19], is a significant cross-sectional survey. Using a stratified, multistage sampling approach, extensive data on a wide range of nutritional, dietary, and health conditions across the population are gathered. The interview and physical examination data are accessible for public download from the official website (https://www.cdc.gov/nchs/nhanes). The NCHS Ethical Review Board approved the study, and informed consent was obtained from all participants.

Participants were selected from 10 cycles of the NHANES survey conducted between 1999 and 2018, encompassing 101,316 individuals. The exclusion criteria included participants under 20 years of age (*n* = 46,235) and those without MetS (*n* = 36,582), resulting in a final cohort of 18,499 adult participants with MetS. Further exclusions were applied to those with missing data for components of the body fat anthropometric indices (AIs) (*n* = 9,533) and those assigned a fasting subsample weight value of “0” (*n* = 581). After those who did not complete follow-up (*n* = 6) were excluded, a total of 8,379 participants remained for analysis (Figure S1).

### Assessment of body fat AIs

Blood samples were obtained from the participants and assessed for biochemical markers, such as fasting blood glucose (FBG), total cholesterol (TC), TG, low-density lipoprotein cholesterol (LDL-C), and HDL-C, according to standardized criteria. Height, weight, and waist circumference (WC) were measured according to established protocols. BMI was calculated as weight (kg) divided by height squared (m²). The WHtR was derived by dividing WC (cm) by height (cm). The calculation methods for various body fat AIs, including the ABSI, BRI, CMI, VAI, WTI, LAP, AIP, and TyG [20, 21], are detailed in Table S1.

### Assessment of MetS and mortality

MetS was defined according to the National Cholesterol Education Program Adult Treatment Panel III (NCEP ATP III) criteria, which require the presence of three or more of at least three of the following conditions: central obesity, elevated FBG, high TG, low HDL-C, and high blood pressure [22].

The mortality data in the NHANES are linked to the National Death Index, with follow-up ending on December 31, 2019. All-cause mortality was defined as death from any cause during the follow-up period, identified via the International Classification of Diseases, 10th Revision (ICD-10) codes [23]. Cardiocerebrovascular disease (CCD) mortality encompasses deaths related to ischemic heart disease, cerebrovascular conditions, and other atherosclerotic diseases (ICD-10 codes I00–I09, I11, I13, I20–I51, I60–I69).

### Covariates

Demographic data, including age, sex (male or female), race/ethnicity (Mexican American, other Hispanic, non-Hispanic white, non-Hispanic black, or other), and education level (below high school, high school, or above high school), were extracted from the NHANES database. Income was assessed via the poverty income ratio (PIR) and categorized into three groups: ≤1.0, 1.1–3.0, and > 3.0 [24]. Lifestyle factors considered included drinking status (nondrinker, low-to-moderate drinker, or heavy drinker), smoking status (never, former, or current smoker), and physical activity (PA) levels (inactive, insufficiently active, or active) [25, 26]. Dietary quality was evaluated with the Healthy Eating Index (HEI), which is ranked into quartiles, whereas comorbidity burden was measured by the Charlson Comorbidity Index (CCI), which is treated as a continuous variable. Detailed definitions for PIR, smoking and drinking status, PA levels, HEI, and CCI are outlined in the supplementary methodology section.

### Statistical analysis

Descriptive statistics for demographics were calculated by sampling weights. Continuous variables with a normal distribution are reported as the means ± standard errors (SEs), whereas those without a normal distribution are presented as medians [interquartile ranges]. Categorical data are shown as counts (percentages). Comparisons of continuous variables were performed via Student’s t test for normally distributed data and the Mann‒Whitney U test for nonnormally distributed data. Categorical variables were compared via the chi-square test. Spearman’s correlation was applied to assess relationships among body fat indices. All analyses were conducted via R (version 4.2.0), with significance defined as *P* < 0.05.

The effectiveness of body fat AIs in predicting all-cause and CCD mortality in individuals with MetS was assessed via the random survival forest (RSF) model. Kaplan‒Meier survival curves and log-rank tests were used to evaluate survival differences between groups. Cox proportional hazards regression was used to analyze the relationships between body fat indices and mortality. Restricted cubic spline (RCS) curves were used to explore potential nonlinear associations. The predictive accuracy of these indices for mortality was further evaluated via receiver operating characteristic (ROC) curves.

Stratified analyses were performed to evaluate how subgroup variables—such as age, sex, race, education level, family PIR, smoking and drinking status, PA, and HEI—affect these associations. To address potential reverse causality bias, additional Cox regression analyses were conducted, excluding participants who either died within the first year of follow-up or had a history of CVD or cancer at baseline.

## Results

### Baseline characteristics of the participants

Table 1 provides an overview of the demographic and clinical characteristics of the study participants. The distribution of participants by age group was as follows: 19.73% aged 20–39 years, 42.04% aged 40–59 years, and 38.23% aged ≥ 60 years. Females accounted for 49.71% of the participants, and males accounted for 50.29%. Among patients with MetS who died from all causes, there was a greater prevalence of older age, non-Hispanic White ethnicity, lower educational attainment, moderate economic status, former smoking history, nondrinker status, and lower PA levels. Notably, ABSI levels were significantly greater in individuals with MetS who died from all causes (*P* < 0.05). Table S2 presents the baseline characteristics of participants with MetS according to quartiles of ABSI levels.

**Table 1:** Baseline characteristics of the metabolic syndrome population according to mortality in NHANES 1999–2018

Characteristics	Total (n = 8379)	All-cause Mortality		*P* value
		No (n = 6681)	Yes (n = 1698)	
Age, years				< 0.001
20–39	1367(19.73)	1335(22.83)	32(3.27)	
40–59	2909(42.04)	2644(45.70)	265(22.58)	
≥ 60	4103(38.23)	2702(31.47)	1401(74.14)	
Sex, %				0.100
Female	4252(49.71)	3483(50.15)	769(47.37)	
Male	4127(50.29)	3198(49.85)	929(52.63)	
Race/ethnicity, %				< 0.001
Mexican American	1564(8.04)	1325(8.82)	239(3.88)	
Other Hispanic	764(5.25)	684(5.66)	80(3.09)	
Non-Hispanic White	3922(70.86)	2862(69.20)	1060(79.66)	
Non-Hispanic Black	1465(9.55)	1196(9.55)	269(9.53)	
Other race	664(6.30)	614(6.77)	50(3.84)	
Education level, %				< 0.001
Below high school	2593(20.50)	1940(18.78)	653(29.65)	
High school	2052(27.45)	1584(26.83)	468(30.74)	
Above high school	3734(52.05)	3157(54.39)	577(39.61)	
Family PIR, %				< 0.001
≤ 1.0	1766(14.42)	1415(14.30)	351(15.05)	
1.1–3.0	3764(39.71)	2889(37.84)	875(49.62)	
> 3.0	2849(45.88)	2377(47.86)	472(35.34)	
Smoking status, %				< 0.001
Never smoker	4161(48.44)	3496(50.61)	665(36.93)	
Former smoker	2586(30.95)	1880(29.21)	706(40.21)	
Current smoker	1632(20.61)	1305(20.18)	327(22.86)	
Drinking status, %				< 0.001
Nondrinker	2229(22.32)	1730(21.26)	499(27.97)	
Low-to-moderate drinker	5546(69.16)	4478(70.38)	1068(62.64)	
Heavy drinker	604(8.52)	473(8.36)	131(9.38)	
Physical activity, %				< 0.001
Inactive	2707(27.40)	1955(24.66)	752(41.93)	
Insufficiently active	3081(40.13)	2523(41.14)	558(34.76)	
Active	2591(32.47)	2203(34.19)	388(23.32)	
Healthy eating index	49.05(39.85,58.38)	48.59(39.46,58.17)	50.67(41.57,59.44)	< 0.001
Charlson comorbidity index	1.41(0.03)	1.25(0.03)	2.23(0.06)	< 0.001
Waist circumference, cm	106.50(98.00,116.90)	106.60(98.20,116.90)	106.00(96.80,116.30)	0.040
Weight, kg	89.40(77.30,104.20)	90.50(78.50,104.90)	83.60(71.10, 99.70)	< 0.001
Height, cm	168.60(161.00,176.50)	168.80(161.30,176.60)	167.50(159.40,175.80)	< 0.001
Body mass index, kg/m^2^	30.98(27.60,35.56)	31.20(27.90,35.80)	29.55(26.14,34.21)	< 0.001
HDL-C, mg/dL	1.14(0.96,1.34)	1.13(0.96,1.32)	1.16(0.98,1.42)	< 0.001
Triglyceride, mg/dL	159.00(109.00,217.00)	159.00(108.00,217.00)	162.00(110.00,222.00)	0.320
Fasting plasma glucose, mg/dL	107.80(101.00,121.00)	107.00(101.00,119.00)	111.40(102.30,130.60)	< 0.001
Body fat AIs				
ABSI	0.08(0.08,0.09)	0.08(0.08,0.09)	0.09(0.08,0.09)	< 0.001
BRI	6.11(4.98,7.70)	6.09(4.98,7.70)	6.17(4.99,7.70)	0.880
CMI	0.23(0.16,0.31)	0.23(0.16,0.31)	0.23(0.16,0.31)	0.120
VAI	2.65(1.69,4.02)	2.65(1.70,3.98)	2.62(1.65,4.09)	0.500
WTI	9.05(8.68,9.38)	9.04(8.69,9.38)	9.07(8.66,9.38)	0.950
LAP	80.22(52.87,117.89)	80.60(53.36,117.77)	77.40(51.10,118.81)	0.150
AIP	3.62(2.26,5.39)	3.63(2.28,5.43)	3.51(2.17,5.32)	0.290
TyG	9.07(8.72,9.43)	9.07(8.71,9.43)	9.12(8.75,9.48)	< 0.001
CCD mortality, %				< 0.001
No	7811(94.96)	6681(100.00)	1130(68.16)	
Yes	568(5.04)	0(0.00)	568(31.84)	
Follow-up time, years	8.50(4.25,13.33)	8.75(4.33,13.83)	7.42(4.00,11.42)	< 0.001

Abbreviations: PIR, poverty income ratio; HDL-C, high-density lipoprotein cholesterol. Ais, anthropometric indices; ABSI, body shape index; BRI, body roundness index; CMI, cardiometabolic index; VAI, visceral adiposity index; WTI, waist triglyceride index; LAP, lipid accumulation product; AIP, atherogenic index of plasma; TyG, triglyceride‒glucose index; CCD, cardio-cerebrovascular disease

Normally distributed continuous variables are described as the means ± SEs, and continuous variables without a normal distribution are presented as medians [interquartile ranges]. Sampling weights were applied for calculation of demographic descriptive statistics; N reflects the study sample, while percentages reflect the survey-weighted data

### The link between ABSI and all-cause mortality in individuals with MetS

**Fig. 1:**
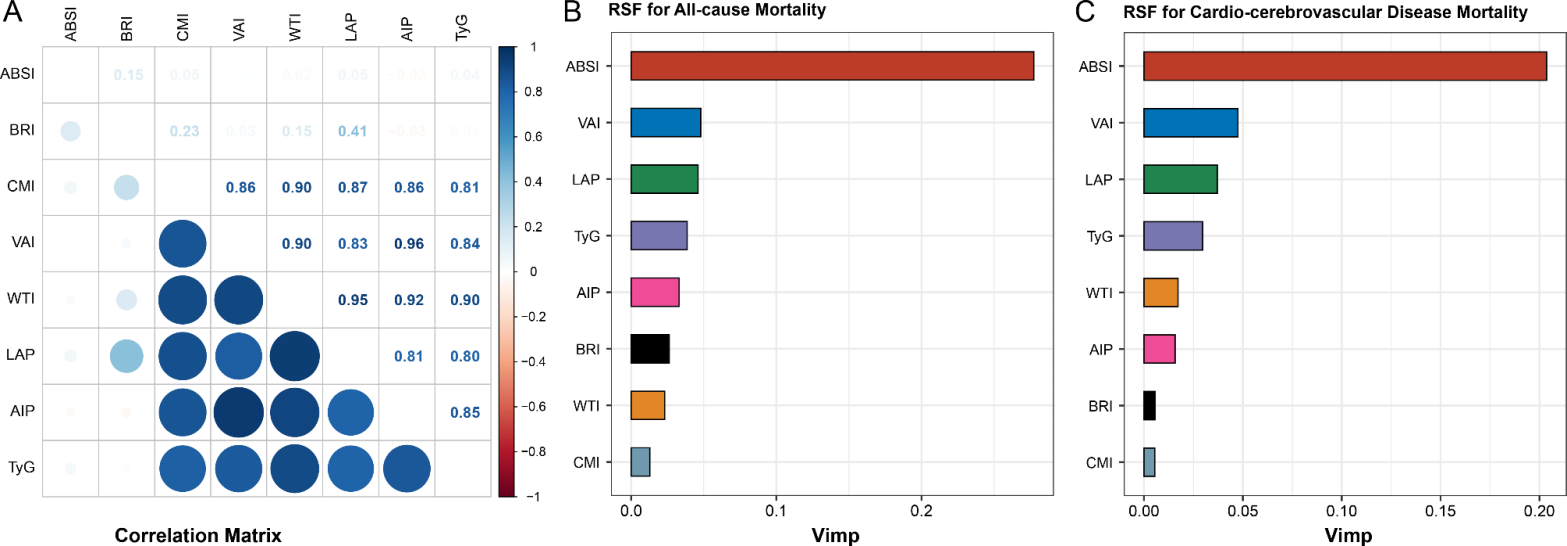
Prognostic significance of body fat AIs. **A**: Spearman correlation coefficients among various body fat AIs. **B**: Random survival forest (RSF) analysis evaluating the predictive value of body fat AIs for all-cause mortality in individuals with MetS. **C**: RSF analysis assessing the predictive value of body fat AIs for CCD mortality in individuals with MetS

**Fig. 2:**
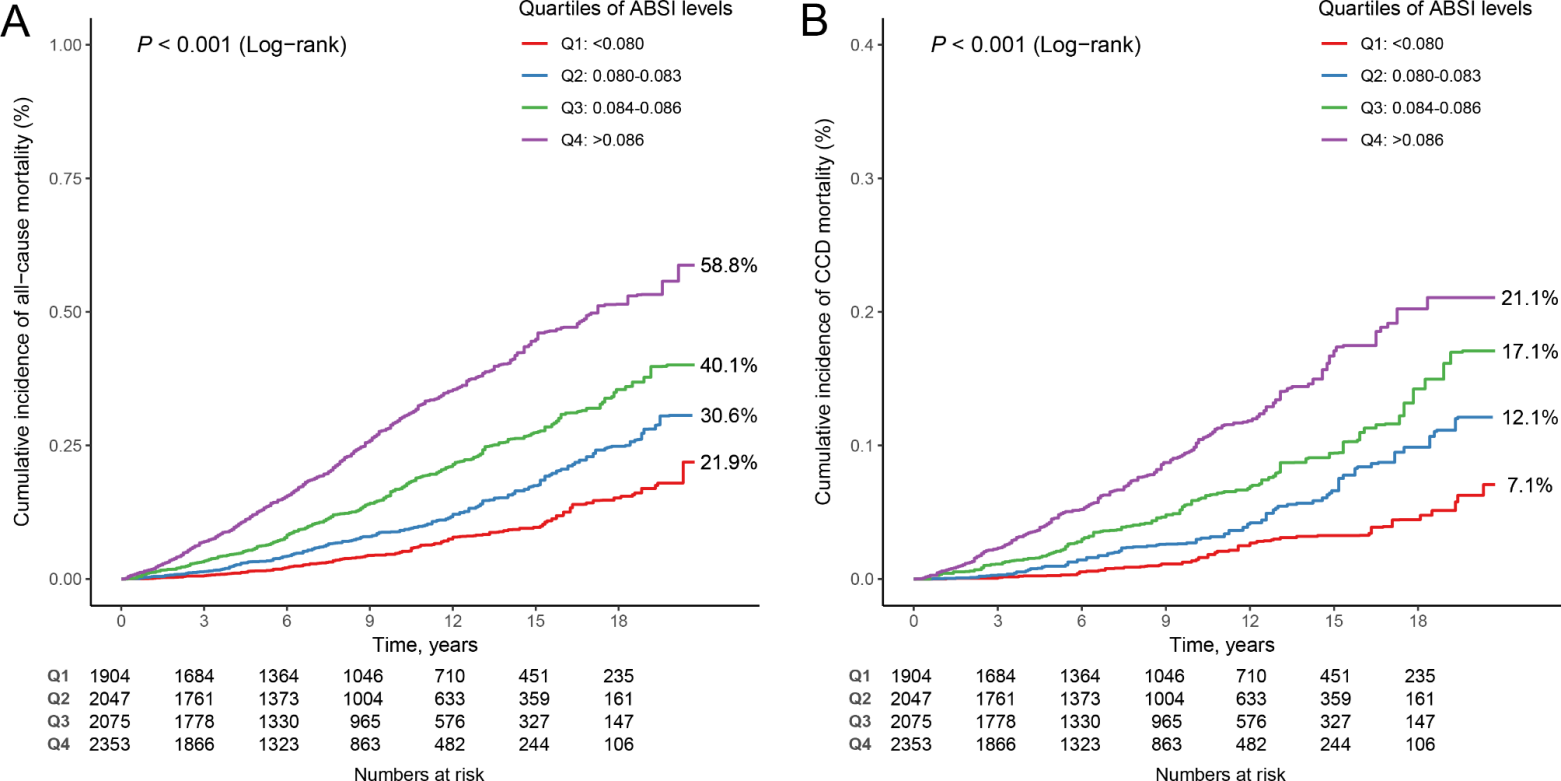
K‒M survival curves for quartiles of ABSI and mortality (**A**: all-cause mortality; **B**: CCD mortality) in individuals with MetS

The correlations among body fat AIs are depicted in Fig. 1A. The RSF method revealed that the ABSI demonstrated the strongest ability to predict all-cause mortality in individuals with MetS (Fig. 1B). Over a median follow-up period of 8.50 years, there were 1,698 deaths from all causes. Kaplan‒Meier survival curves illustrating the association between ABSI and all-cause mortality in individuals with MetS are presented in Fig. 2A. Individuals with MetS with high ABSI values had the highest risk of all-cause mortality (log-rank test *P* < 0.001).

**Fig. 3:**
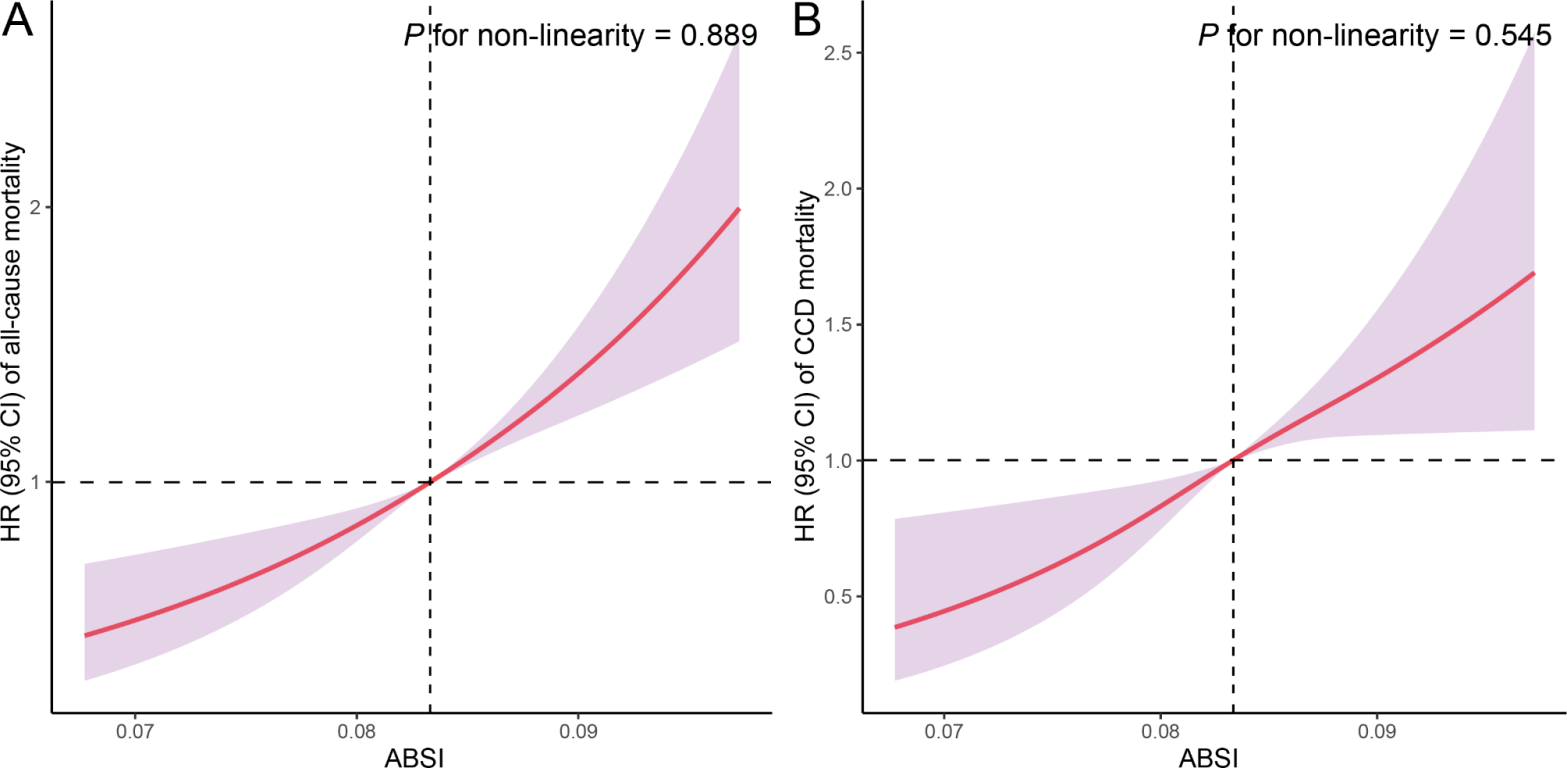
Restricted cubic spline (RCS) analysis with multivariate-adjusted associations of ABSI and mortality (**A**: all-cause mortality; **B**: CCD mortality) in individuals with MetS. The models are adjusted for age (20–39, 40–59, or ≥ 60), sex (male or female), race/ethnicity (Mexican American, other Hispanic, non-Hispanic White, non-Hispanic Black, or other race), education level (below high school, high school, or above high school), family PIR (≤ 1.0, 1.1–3.0, or > 3.0), smoking status (never smoker, former smoker, or current smoker), drinking status (nondrinker, low–to–moderate drinker, or heavy drinker), PA (inactive, insufficiently active, or active), HEI (in quartiles), and CCI (continous)

In both the crude model and model 1 adjustments, all-cause mortality was significantly elevated among individuals with MetS with high ABSI values compared with those with lower ABSI values (Table 2). Specifically, individuals in the highest quartile of ABSI had an increased risk of all-cause mortality compared with those in the lowest quartile (HR = 1.773 [1.419–2.215]). RCS analyses revealed a linear relationship between ABSI and all-cause mortality in individuals with MetS (*P* for nonlinearity = 0.889) (Fig. 3A). Further analyses examining the associations between other body fat AIs and all-cause mortality in individuals with MetS are detailed in Table S3 and Figure S2.

**Table 2:** HR (95% CIs) of mortality according to quartiles of ABSI among individuals with metabolic syndrome in NHANES 1999–2018

	Quartiles of ABSI levels				*P*_trend_
	< 0.080	0.080–0.083	0.084–0.086	> 0.086	
All-cause Mortality					
Crude	1 [Reference]	1.719 (1.350–2.188)	2.892 (2.312–3.618)	5.339 (4.335–6.575)	< 0.001
Model 1	1 [Reference]	1.348 (1.057–1.719)	1.743 (1.395–2.177)	2.402 (1.927–2.993)	< 0.001
Model 2	1 [Reference]	1.249 (0.972–1.606)	1.546 (1.237–1.933)	1.773 (1.419–2.215)	< 0.001
Cardio-cerebrovascular Disease Mortality					
Crude	1 [Reference]	2.008 (1.417–2.844)	3.236 (2.287–4.579)	5.597 (4.187–7.483)	< 0.001
Model 1	1 [Reference]	1.513 (1.078–2.123)	1.795 (1.270–2.537)	2.287 (1.670–3.130)	< 0.001
Model 2	1 [Reference]	1.438 (1.021–2.024)	1.629 (1.151–2.307)	1.735 (1.267–2.375)	0.004

Abbreviations: HR, hazard ratio; CI, confidence interval; ABSI, a body shape index

Model 1 was adjusted for age (20–39, 40–59, or ≥ 60), sex (male or female), race/ethnicity (Mexican American, other Hispanic, non-Hispanic White, non-Hispanic Black, or other race); Model 2 was adjusted for model 1 plus education level (below high school, high school, or above high school), family PIR (≤ 1.0, 1.1–3.0, or > 3.0), smoking status (never smoker, former smoker, or current smoker), drinking status (nondrinker, low-to-moderate drinker, or heavy drinker), PA (inactive, insufficiently active, or active), HEI (in quartiles), and CCI (continous)

### The link between ABSI and CCD mortality in individuals with MetS

RSF analysis revealed that ABSI exhibited the strongest predictive capacity for CCD mortality in individuals with MetS (Fig. 1C). Over a median follow-up of 8.50 years, 568 deaths from CCD were recorded. Kaplan‒Meier survival curves illustrating the association between ABSI and CCD mortality in individuals with MetS are depicted in Fig. 2B. Individuals with MetS with elevated ABSI values presented the highest risk of CCD mortality (log-rank test *P* < 0.001). According to both the crude model and model 1 adjustments, CCD mortality was significantly elevated among individuals with MetS with high ABSI values compared with those with lower ABSI values (Table 2). Specifically, individuals in the highest quartile of ABSI had an increased risk of CCD mortality compared with those in the lowest quartile (HR = 1.735 [1.267–2.375]). RCS analyses revealed a linear relationship between ABSI and CCD mortality in individuals with MetS (*P* for nonlinearity = 0.545) (Fig. 3B). Further analyses exploring the associations between additional body fat AIs and CCD mortality in individuals with MetS are detailed in Table S4 and Figure S3.

### The predictive value of the ABSI for mortality

**Fig. 4:**
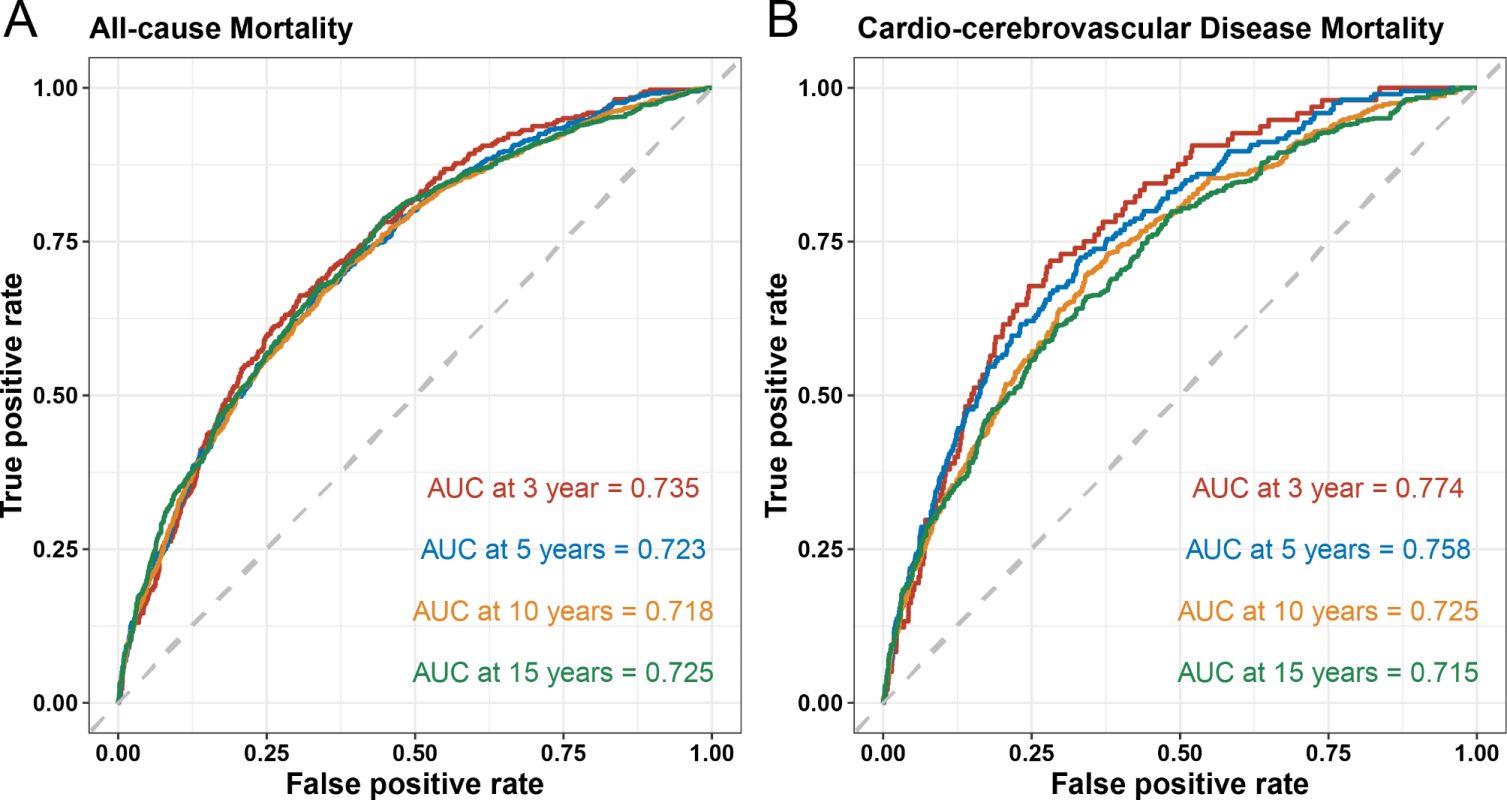
Predictive value of the time-dependent ROC assessment of the ABSI for 3-, 5-, 10-, and 15-year mortality (**A**: all-cause mortality; **B**: CCD mortality) in individuals with MetS

According to the ROC curve analysis, the ABSI demonstrated robust predictive performance for both all-cause mortality in individuals with MetS (AUC at 3, 5, 10, and 15 years = 0.735, 0.723, 0.718, and 0.725) (Fig. 4A) and CCD mortality (AUC at 3, 5, 10, and 15 years = 0.774, 0.758, 0.725, and 0.715) (Fig. 4B). Compared with other body fat AIs, the ABSI greatly improved the predictive value of both outcomes (Figure S4-5). In addition, Table S5 summarizes the improvement in risk prediction for all-cause and CCD mortality by adding the ABSI to a fully adjusted model, which yielded significantly higher AUC values across all time points evaluated.

### Stratified analyses and sensitivity analyses

Stratified and sensitivity analyses were conducted to examine the associations between quartiles of ABSI and the risk of all-cause mortality among individuals with MetS, stratified by age, sex, race, education level, family PIR, smoking status, drinking status, PA, and HEI (Tables 3 and 4). Overall, the association between ABSI and mortality remained consistent across all subgroups.

**Table 3:** Stratified analyses of the associations between quartiles of ABSI levels and the risk of all-cause mortality among individuals with metabolic syndrome in NHANES 1999–2018

Subgroups	*N*	Quartiles of ABSI levels				*P*-int
		< 0.080	0.080–0.083	0.084–0.086	> 0.086	
Age						0.868
20–39	1367	1 [Reference]	1.151 (0.409–3.239)	2.328 (0.708–7.656)	1.344 (0.304–5.940)	
40–59	2909	1 [Reference]	1.227 (0.816–1.843)	1.757 (1.173–2.633)	1.811 (1.123–2.921)	
≥ 60	4103	1 [Reference]	1.283 (0.946–1.742)	1.442 (1.103–1.883)	1.897 (1.460–2.465)	
Sex						0.444
Female	4252	1 [Reference]	1.338 (0.877–2.041)	1.762 (1.175–2.641)	2.311 (1.556–3.432)	
Male	4127	1 [Reference]	1.312 (0.984–1.750)	1.562 (1.188–2.055)	1.712 (1.334–2.196)	
Race/ethnicity						0.467
Mexican American	1564	1 [Reference]	1.108 (0.608–2.017)	1.456 (0.767–2.765)	2.079 (1.130–3.826)	
Other Hispanic	764	1 [Reference]	1.407 (0.334–5.927)	1.531 (0.402–5.832)	1.979 (0.519–7.551)	
Non-Hispanic White	3922	1 [Reference]	1.239 (0.907–1.693)	1.575 (1.201–2.065)	1.859 (1.435–2.409)	
Non-Hispanic Black	1465	1 [Reference]	1.236 (0.831–1.841)	1.402 (0.927–2.123)	2.271 (1.556–3.317)	
Other race	664	1 [Reference]	1.933 (0.563–6.631)	1.760 (0.426–7.270)	1.636 (0.468–5.726)	
Education level						0.433
Below high school	2593	1 [Reference]	1.123 (0.729–1.730)	1.594 (1.118–2.273)	1.762 (1.279–2.428)	
High school	2052	1 [Reference]	1.754 (1.119–2.749)	1.811 (1.184–2.769)	2.672 (1.761–4.054)	
Above high school	3734	1 [Reference]	1.111 (0.784–1.576)	1.401 (0.966–2.032)	1.599 (1.085–2.358)	
Family PIR						0.388
≤ 1.0	1766	1 [Reference]	1.445 (0.836–2.496)	2.063 (1.245–3.417)	2.385 (1.464–3.886)	
1.1–3.0	3764	1 [Reference]	1.070 (0.760–1.507)	1.489 (1.056–2.101)	1.630 (1.175–2.261)	
> 3.0	2849	1 [Reference]	1.559 (1.077–2.255)	1.516 (1.035–2.221)	2.188 (1.516–3.156)	
Smoking status						0.078
Never smoker,	4161	1 [Reference]	1.411 (1.014–1.965)	1.755 (1.300–2.370)	1.861 (1.369–2.530)	
Former smoker	2586	1 [Reference]	1.320 (0.869–2.006)	1.314 (0.851–2.030)	2.179 (1.408–3.373)	
Current smoker	1632	1 [Reference]	1.020 (0.628–1.658)	1.639 (0.975–2.757)	1.611 (1.006–2.579)	
Drinking status						0.278
Nondrinker,	2229	1 [Reference]	1.322 (0.920–1.898)	1.463 (1.010–2.120)	1.688 (1.187-2.400)	
Low-to-moderate drinker	5546	1 [Reference]	1.324 (0.950–1.846)	1.718 (1.240–2.380)	2.179 (1.612–2.946)	
Heavy drinker	604	1 [Reference]	1.030 (0.455–2.331)	1.238 (0.577–2.656)	1.283 (0.600-2.743)	
Physical activity						0.838
Inactive	2707	1 [Reference]	1.094 (0.781–1.534)	1.334 (0.971–1.833)	1.657 (1.230–2.233)	
Insufficiently active	3081	1 [Reference]	1.321 (0.887–1.968)	1.791 (1.186–2.704)	2.171 (1.405–3.357)	
Active	2591	1 [Reference]	1.594 (0.982–2.588)	1.753 (1.087–2.829)	2.170 (1.366–3.445)	
HEI						0.361
Quartile 1	2095	1 [Reference]	0.920 (0.590–1.434)	1.413 (0.923–2.163)	1.405 (0.916–2.155)	
Quartile 2	2095	1 [Reference]	1.654 (1.033–2.649)	1.946 (1.306–2.899)	2.018 (1.312–3.105)	
Quartile 3	2094	1 [Reference]	1.367 (0.893–2.094)	1.348 (0.852–2.132)	2.215 (1.458–3.365)	
Quartile 4	2095	1 [Reference]	1.244 (0.737-2.100)	1.665 (1.052–2.636)	2.145 (1.325–3.474)	

Abbreviations: *P-int*, *P* for interaction; HR, hazard ratio; CI, confidence interval; ABSI, a body shape index

The data are presented as HRs (95% CIs) unless otherwise indicated. Analyses were adjusted for age (20–39, 40–59, or ≥ 60), sex (male or female), race/ethnicity (Mexican American, other Hispanic, non-Hispanic White, non-Hispanic Black, or other races), education level (below high school, high school, or above high school), family PIR (≤ 1.0, 1.1–3.0, or > 3.0), smoking status (never smoker, former smoker, or current smoker), drinking status (nondrinker, low–to–moderate drinker, or heavy drinker), PA (inactive, insufficiently active, or active), HEI (in quartiles), and CCI (continous) when they were not the strata variables

**Table 4:** Stratified analyses of the associations between quartiles of ABSI levels and the risk of cardio-cerebrovascular disease mortality among individuals with metabolic syndrome in NHANES 1999–2018

Subgroups	*N*	Quartiles of ABSI levels				*P*-int
		< 0.080	0.080–0.083	0.084–0.086	> 0.086	
Age						0.092
20–39	1367	1 [Reference]	0.278 (0.058–1.330)	0.855 (0.100-7.295)	0.466 (0.016–13.739)	
40–59	2909	1 [Reference]	2.872 (1.461–5.645)	4.779 (2.305–9.912)	3.861 (1.797–8.297)	
≥ 60	4103	1 [Reference]	1.302 (0.851–1.992)	1.279 (0.883–1.854)	1.618 (1.106–2.368)	
Sex						0.995
Female	4252	1 [Reference]	1.540 (0.838–2.831)	1.739 (0.849–3.559)	2.011 (1.030–3.925)	
Male	4127	1 [Reference]	1.450 (0.908–2.317)	1.661 (1.070–2.577)	1.816 (1.259–2.619)	
Race/ethnicity						0.595
Non-Hispanic White	3922	1 [Reference]	1.398 (0.912–2.141)	1.638 (1.072–2.502)	1.697 (1.112–2.589)	
Non-Hispanic Black	1465	1 [Reference]	1.448 (0.850–2.464)	1.329 (0.694–2.547)	2.508 (1.385–4.541)	
Other	2992	1 [Reference]	2.761 (0.938–8.123)	3.562 (1.373–9.241)	4.228 (1.588–11.256)	
Education level						0.529
Below high school	2593	1 [Reference]	0.956 (0.484–1.889)	1.316 (0.782–2.213)	1.308 (0.805–2.124)	
High school	2052	1 [Reference]	2.382 (1.053–5.390)	2.583 (1.064–6.269)	3.143 (1.403–7.044)	
Above high school	3734	1 [Reference]	1.569 (0.861–2.861)	1.604 (0.790–3.255)	1.878 (0.939–3.756)	
Family PIR						0.210
≤ 1.0	1766	1 [Reference]	3.416 (1.319–8.847)	3.096 (1.256–7.633)	3.823 (1.640–8.911)	
1.1–3.0	3764	1 [Reference]	0.892 (0.525–1.516)	1.251 (0.751–2.085)	1.307 (0.822–2.079)	
> 3.0	2849	1 [Reference]	2.261 (1.203–4.249)	2.246 (1.074–4.698)	2.648 (1.350–5.195)	
Smoking status						0.424
Never smoker,	4161	1 [Reference]	1.629 (0.968–2.742)	2.125 (1.171–3.857)	2.251 (1.309–3.872)	
Former smoker	2586	1 [Reference]	1.620 (0.930–2.820)	1.230 (0.717–2.110)	1.608 (0.936–2.761)	
Current smoker	1632	1 [Reference]	0.784 (0.354–1.737)	1.252 (0.506–3.098)	1.476 (0.710–3.070)	
Drinking status						0.068
Nondrinker,	2229	1 [Reference]	1.525 (0.835–2.786)	1.112 (0.633–1.952)	1.279 (0.763–2.145)	
Low-to-moderate drinker	5546	1 [Reference]	1.758 (1.019–3.033)	2.341 (1.383–3.960)	2.537 (1.563–4.119)	
Heavy drinker	604	1 [Reference]	0.545 (0.180–1.648)	0.704 (0.219–2.261)	1.052 (0.460–2.407)	
Physical activity						0.434
Inactive	2707	1 [Reference]	1.333 (0.803–2.211)	1.303 (0.762–2.227)	1.564 (0.956–2.557)	
Insufficiently active	3081	1 [Reference]	2.239 (1.018–4.924)	2.094 (0.945–4.638)	2.607 (1.231–5.521)	
Active	2591	1 [Reference]	0.909 (0.437–1.891)	1.827 (0.878–3.804)	1.834 (0.935–3.597)	
HEI						0.509
Quartile 1	2095	1 [Reference]	1.025 (0.473–2.224)	1.006 (0.455–2.228)	0.942 (0.453–1.958)	
Quartile 2	2095	1 [Reference]	2.192 (1.032–4.656)	1.768 (0.905–3.453)	2.169 (1.036–4.539)	
Quartile 3	2094	1 [Reference]	1.568 (0.784–3.138)	1.637 (0.730–3.667)	2.519 (1.227–5.174)	
Quartile 4	2095	1 [Reference]	1.191 (0.577–2.458)	2.261 (1.200-4.263)	2.116 (1.063–4.209)	

Abbreviations: *P-int*, *P* for interaction; HR, hazard ratio; CI, confidence interval; ABSI, a body shape index

The data are presented as HRs (95% CIs) unless otherwise indicated. Analyses were adjusted for age (20–39, 40–59, or ≥ 60), sex (male or female), race/ethnicity (Mexican American, other Hispanic, non-Hispanic White, non-Hispanic Black, or other races), education level (below high school, high school, or above high school), family PIR (≤ 1.0, 1.1–3.0, or > 3.0), smoking status (never smoker, former smoker, or current smoker), drinking status (nondrinker, low–to–moderate drinker, or heavy drinker), PA (inactive, insufficiently active, or active), HEI (in quartiles), and CCI (continous) when they were not the strata variables

Furthermore, sensitivity analyses excluding participants who died within two years of follow-up confirmed the robustness of the association between ABSI and all-cause mortality in individuals with MetS (Table S6). Additionally, after excluding participants with a history of cancer or CVD at baseline, the results remained stable (Tables S7-8).

## Discussion

A total of 8,379 individuals with MetS were included in this study. Among all eight indices examined, the ABSI exhibited the highest predictive capacity for both all-cause mortality and CCD mortality in individuals with MetS. High ABSI values in individuals with MetS were associated with increased risks of all-cause mortality (HR = 1.773 [1.419–2.215]) and CCD mortality (HR = 1.735 [1.267–2.375]) compared with those in the lowest quartile. Furthermore, the association between ABSI and mortality in individuals with MetS was consistent across various stratification characteristics.

MetS represents a cluster of clinical conditions characterized by insulin resistance and multiple metabolic abnormalities [27]. These factors collectively increase the risk of CVD, diabetes, and other health issues [7]. The pathogenesis of MetS is multifactorial and involves genetic predispositions, environmental factors, and lifestyle choices, such as poor dietary habits, physical inactivity, and chronic psychological stress, which may contribute to its onset or exacerbation [28]. Excessive accumulation of abdominal fat, a hallmark of MetS, poses significant health risks [29]. The ABSI, a novel body fat anthropometric index, provides a more precise assessment of the relationship between body fat distribution and health risks by integrating WC, height, and weight [9]. Unlike traditional BMI, ABSI accounts for variations in WC relative to height, thereby offering improved predictive accuracy for health-related risks [30]. Elevated ABSI values typically indicate increased abdominal fat accumulation, which is closely associated with core pathological mechanisms of MetS, including insulin resistance and chronic inflammation.

Currently, the association between ABSI and MetS remains contentious. Sugiura et al. reported that ABSI was independently associated with MetS diagnosis on the basis of WC or visceral fat area [17]. Conversely, on the basis of ROC analysis, Haghighatdoost et al. concluded that the ABSI was a weak predictor of MetS [31]. Stefanescu et al. reported that the ABSI performed less effectively than the BRI in predicting MetS [32], whereas a Chinese study indicated that the TyG score was a more robust predictor of MetS than the ABSI score was [33]. Despite these findings, the ABSI has a distinct advantage in predicting outcomes related to metabolic disorder-associated diseases [30, 34]. To ascertain the optimal predictor of mortality risk in individuals with MetS, we conducted further analyses to explore the correlation between various body fat AIs (ABSI, BRI, CMI, VAI, WTI, LAP, AIP, and TyG) and prognosis among MetS patients. Surprisingly, the ABSI was the strongest predictor of mortality risk in individuals with MetS. Among MetS participants, all-cause mortality increased by 125.0%, 154.2%, and 177.8% in the second, third, and fourth quartiles of ABSI, respectively, compared with the lowest quartile. Additionally, CCD mortality increased by 143.8%, 162.8%, and 173.4% in the second, third, and fourth quartiles of ABSI, respectively, compared with the lowest quartile among MetS participants.

The association between ABSI and mortality has garnered considerable attention. Elevated ABSI often signifies increased abdominal fat accumulation and unfavorable body composition, both of which are significant risk factors for MetS and its associated disorders [35]. First, a high ABSI is strongly associated with the onset of metabolic disorders. Wu et al. demonstrated that ABSI is correlated with insulin resistance in the general adult population and in older adults without diabetes [36]. Christakoudi et al. reported positive associations between ABSI scores and TG and inflammatory marker levels and negative correlations with HDL-C levels [37]. Fundamental research indicates that excessive fat accumulation releases inflammatory cytokines and free fatty acids, disrupting insulin action and promoting insulin resistance [38]. These inflammatory mediators also disrupt lipid metabolism, contributing to dyslipidemia. Second, elevated ABSI is linked to hypertension [39]. Hall et al. concluded that excessive weight gain, particularly visceral fat accumulation, significantly contributes to hypertension [40]. Chronic hypertension compromises cardiovascular structure and function, thereby increasing CVD morbidity and mortality in MetS patients. Additionally, the ABSI serves as a predictor for the development of other metabolic diseases, including diabetes mellitus and nonalcoholic fatty liver disease [41].

The ABSI was the most potent predictor of mortality among the eight body fat AIs evaluated in individuals with MetS. However, as noted by Krakauer [42], the predictive accuracy for mortality risk can be further enhanced by combining body fat AIs with MetS component scores. This approach integrates the strengths of both methods, capturing the distribution of body fat through indices such as ABSI, while also accounting for metabolic abnormalities such as dyslipidemia, hypertension, and hyperglycemia. The combination of these metrics could provide a more comprehensive risk assessment, improving the identification of high-risk individuals. Future research should focus on developing and validating models that incorporate both body fat anthropometric and metabolic parameters, potentially leading to more effective early interventions and better patient outcomes.

The superior performance of the ABSI in predicting mortality among individuals with MetS may be due to its derivation as a power law, similar to BMI. The ABSI adjusts the WC for weight and height, thereby capturing the effect of the WC independent of these factors. This adjustment likely accounts for ABSI’s stronger predictive power than other body fat AIs. Notably, ABSI and the BRI are distinct in that they do not include lipid serum biomarkers. In contrast, indices such as the VAI, AIP, and TyG incorporate lipid markers—TG and HDL-C—which are already part of the MetS criteria. These overlaps may limit the additional predictive value of lipid-based indices for mortality risk, particularly within the MetS context. Additionally, the VAI, which adjusts for height through BMI, performs slightly better among lipid-based indices. Therefore, it is unsurprising that body fat AIs incorporating lipid measures, especially the AIP and TyG, contribute minimally to mortality risk prediction, as shown in the supplementary data.

### Study strengths and limitations

Using a large, representative sample of the U.S. population with long-term follow-up, this study investigated the relationships between eight body fat AIs and mortality in individuals with MetS, identifying the ABSI score as the most effective predictor of all-cause mortality and CCD mortality. While this study identified associations between ABSI and mortality in MetS individuals, several limitations must be acknowledged. First, the data were derived from public databases and confined to the U.S. population. Second, dynamic changes in these indicators were not analyzed. Third, the study did not account for potential influences such as genetic and environmental factors.

## Conclusion

In individuals with MetS, an elevated ABSI was associated with increased risks of all-cause and CCD mortality. Among the eight body fat AIs evaluated, the ABSI emerged as the strongest predictor of mortality in this population. These findings underscore the clinical importance of incorporating ABSI into routine risk assessment for patients with MetS. By identifying those at higher risk more accurately, ABSI can guide more targeted interventions and personalized management strategies, ultimately improving patient outcomes. Future research should focus on integrating ABSI into clinical practice and exploring its potential to refine risk stratification and treatment approaches in this high-risk population.

## Electronic supplementary material

Below is the link to the electronic supplementary material.

Supplementary Material 1

## Data Availability

NHANES data described in this manuscript are available at https://wwwn.cdc.gov/nchs/nhanes/.
