# Supplementary material for: Associations between body fat anthropometric indices and mortality among individuals with metabolic syndrome
Source: Lipids Health Dis. 2024 Sep 27;23:306. doi: 10.1186/s12944-024-02272-0 (PMC11429950; doi:10.1186/s12944-024-02272-0)
Supplement: Supplementary file 1 — Supplementary Material 1 [file 12944_2024_2272_MOESM1_ESM.docx]

**Supplementary Materials**

**Association between anthropometric indexes and mortality among the individuals with metabolic syndrome**

**Figure S1.** Flowchart of the study

**Table S1.** Calculation methods of combination in each body fat anthropometric indices.

**Table S2.** Baseline characteristics of the metabolic syndrome population according to quartiles of ABSI levels in NHANES 1999–2018.

**Table S3.** HR (95% CIs) of all-cause mortality according to quartiles of anthropometric indexes among individuals with metabolic syndrome in NHANES 1999–2018.

**Figure S2.** Restricted cubic spline (RCS) analysis with multivariate-adjusted associations of anthropometric indexes with all-cause mortality among individuals with metabolic syndrome in NHANES 1999–2018.

**Table S4.** HR (95% CIs) of cardio-cerebrovascular disease mortality according to quartiles of anthropometric indexes among individuals with metabolic syndrome in NHANES 1999–2018.

**Figure S3.** Restricted cubic spline (RCS) analysis with multivariate-adjusted associations of anthropometric indexes with cardio-cerebrovascular disease mortality among individuals with metabolic syndrome in NHANES 1999–2018.

**Figure S4.** Predictive value of time-dependent ROC assessment of anthropometric indexes for 3-, 5-, 10-, and 15-year all-cause mortality.

**Figure S5.** Predictive value of time-dependent ROC assessment of anthropometric indexes for 3-, 5-, 10-, and 15-year cardio-cerebrovascular disease mortality.

**Table S5.** Improvement of risk prediction by adding ABSI to fully adjusted model.

**Table S6.** HR (95% CIs) of mortality according to quartiles of ABSI after excluding participants who died within one years of follow-up among individuals with metabolic syndrome in NHANES 1999–2018.

**Table S7.** HR (95% CIs) of mortality according to quartiles of ABSI after excluding participants who had CVD history at baseline among individuals with metabolic syndrome in NHANES 1999–2018.

**Table S8.** HR (95% CIs) of mortality according to quartiles of ABSI after excluding participants who had cancer history at baseline among individuals with metabolic syndrome in NHANES 1999–2018.

**Materials and methods: Other Covariates**

This section provides a comprehensive definition of potential confounding variables including family poverty income ratio (PIR, ≤1.0, 1.1–3.0, or >3.0), smoking status (never, former, or current smoker), drinking status (nondrinker, former drinker, or current drinker), physical activity (inactive, insufficiently active, or active), healthy eating index (HEI), and Charlson comorbidity index (CCI).

***Family poverty income ratio*** Income was assessed using the poverty income ratio (PIR, the ratio of family income divided by a poverty threshold specific for family size using guidelines from the US Department of Health and Human Services) and categorized as ≤1.0, 1.1-3.0 and >3.0 [1].

***Smoking status*** Never smokers were classified as those who reported smoking <100 cigarettes during their lifetime. Those who smoked >100 cigarettes in their lifetime were considered as current smokers, and those who smoked >100 cigarettes and had quit smoking were considered as former smokers [2].

***Drinking status*** Drinking status was classified as nondrinker, low-to-moderate drinker (<2 drinks/day in men and <1 drink/day in women), or heavy drinker (≥2 drinks/day in men and ≥1 drinks/day in women) [2].

***Physical activity*** Physical activity was categorized as inactive group (no leisure-time physical activity), insufficiently active group (leisure time moderate activity 1–5 times per week with MET ranging from 3 to 6 or leisure-time vigorous activity 1–3 times per week with MET >6), or active group (those who had more leisure-time moderate-or-vigorous activity than above) [3].

***Healthy Eating Index*** The Healthy Eating Index (HEI) is a measure calculated from 24-hour dietary recall data to assess diet quality based on the 2015–2020 Dietary Guidelines for Americans (DGA) [4]. It comprises 13 subgroups, with a total possible score of 100. Nine components evaluate adequacy (higher intakes contribute to a higher score) including total fruits, whole fruits, total vegetables, greens and beans, whole grains, dairy, total protein foods, seafood and plant proteins, and fatty acids. The remaining four components assess moderation (lower intakes yield a higher score), covering refined grains, sodium, added sugars, and saturated fats. The HEI offers a comprehensive framework for understanding dietary patterns and their relationship to health outcomes, with scores reflecting adherence to key dietary recommendations.

***Charlson Comorbidity Index*** The Charlson Comorbidity Index (CCI) is a method used to quantify an individual's overall health status by summing the scores assigned to various diseases [5]. It assumes respondents without reported diseases as healthy, assigning a zero value to unreported conditions. This scoring approach follows the methodology established by Zhao et al. in prior research [6], ensuring consistency in the assessment of comorbidities.

**References**

1. **Services USDoHaH. Poverty Guidelines, Research, and Measurement** [<http://aspe.hhs.gov/POVERTY/index.shtml>.]

2. Qiu Z, Chen X, Geng T, Wan Z, Lu Q, Li L, Zhu K, Zhang X, Liu Y, Lin X *et al*: **Associations of Serum Carotenoids With Risk of Cardiovascular Mortality Among Individuals With Type 2 Diabetes: Results From NHANES**. *Diabetes Care* 2022, **45**(6):1453-1461.

3. Beddhu S, Baird BC, Zitterkoph J, Neilson J, Greene T: **Physical activity and mortality in chronic kidney disease (NHANES III)**. *Clin J Am Soc Nephrol* 2009, **4**(12):1901-1906.

4. Kirkpatrick SI, Reedy J, Krebs-Smith SM, Pannucci TE, Subar AF, Wilson MM, Lerman JL, Tooze JA: **Applications of the Healthy Eating Index for Surveillance, Epidemiology, and Intervention Research: Considerations and Caveats**. *J Acad Nutr Diet* 2018, **118**(9):1603-1621.

5. Kim CY, Sivasundaram L, LaBelle MW, Trivedi NN, Liu RW, Gillespie RJ: **Predicting adverse events, length of stay, and discharge disposition following shoulder arthroplasty: a comparison of the Elixhauser Comorbidity Measure and Charlson Comorbidity Index**. *J Shoulder Elbow Surg* 2018, **27**(10):1748-1755.

6. Zhao H, Pan Y, Wang C, Guo Y, Yao N, Wang H, Li B: **The Effects of Metal Exposures on Charlson Comorbidity Index Using Zero-Inflated Negative Binomial Regression Model: NHANES 2011-2016**. *Biol Trace Elem Res* 2021, **199**(6):2104-2111.

**
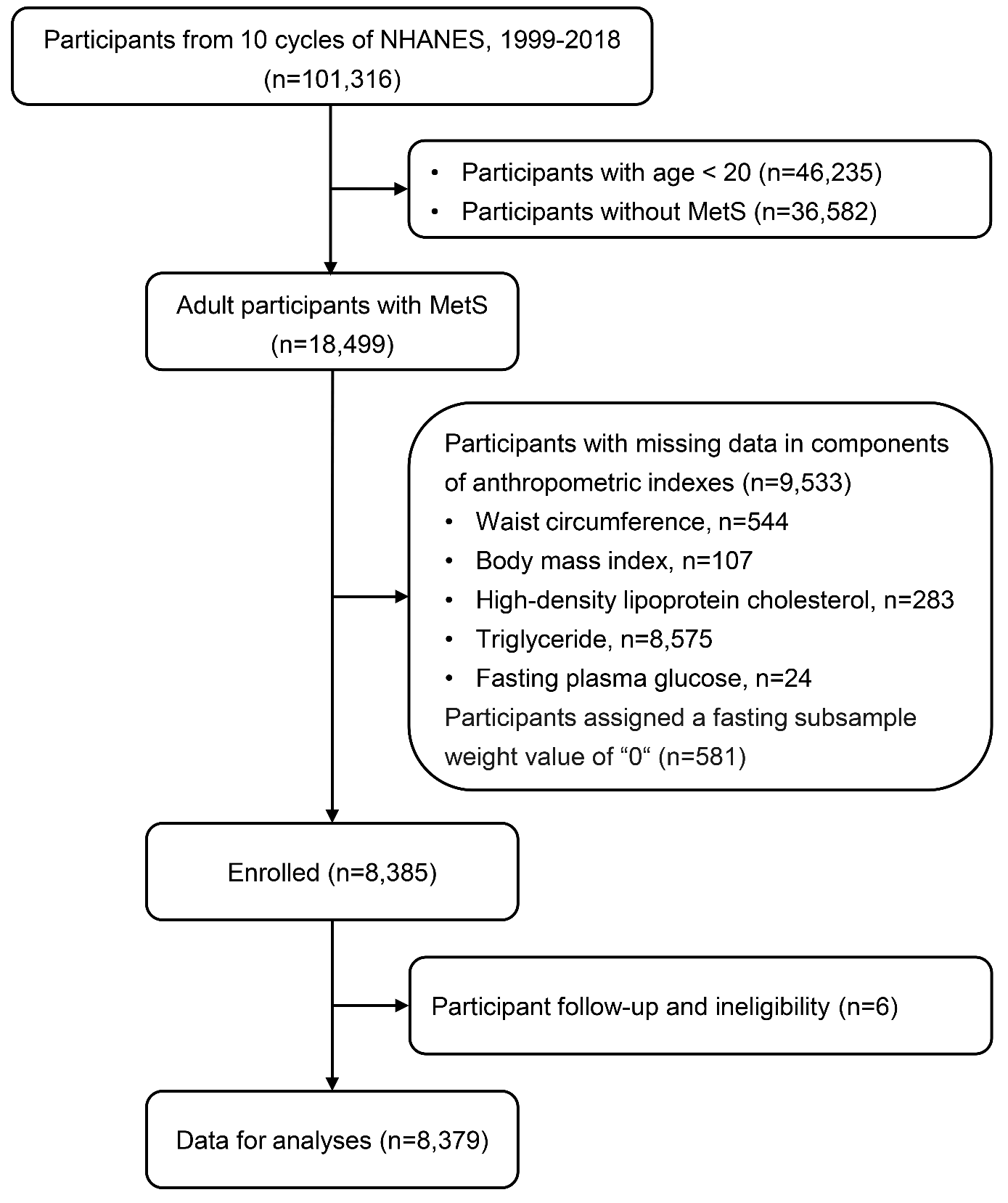
**

**Figure S1.** Flowchart of the study

**Table S1.** Calculation methods of combination in each body fat anthropometric indices.

| Indicators | Definition or calculation formula |
| --- | --- |
| ABSI | WC (cm)/[BMI(kg/m^2^)^2/3^ - height (m)^1/2^] |
| BRI | 364.2-365.5×(1-[WC(m)/2π]^2^/[0.5×height(m)]^2^)×0.5 |
| CMI | TG(mmol/L)/HDL-C(mmol/L)×WHtR |
| VAI | [WC (cm)/39.68+(1.88×BMI(kg/m^2^))]×(TG(mmol/L)/1.03)×(1.31/HDL−C(mmol/L)) for male and [ WC(cm)/36.58+(1.89×BMI(kg/m^2^))] (TG(mmol/L)/0.81)×(1.52/HDL−C(mmol/L)) for female |
| WTI | Ln [TG(mg/dL)×WC(cm)/2] |
| LAP | [WC (cm)−65]×TG(mmol/L) for male and [WC (cm)−58]× TG(mmol/L) for female |
| AIP | Log(TG/HDL-C) |
| TyG | Ln [TG(mg/dL)× FBG (mg/dL)/2] |

Abbreviations: WC, waist circumference; BMI, body mass index; TG, triglyceride; HDL-C, high-density lipoprotein cholesterol; WHtR, waist-to-height ratio; FBG, fasting blood glucose; ABSI, a body shape index; BRI, body roundness index; CMI, cardiometabolic index; VAI, visceral adiposity index; WTI, waist triglyceride index; LAP, lipid accumulation product; AIP, atherogenic index of plasma; TyG, triglyceride-glucose index.

**Table S2.** Baseline characteristics of the metabolic syndrome population according to quartiles of ABSI levels in NHANES 1999–2018.

| Characteristics | Quartiles of ABSI levels | | | | *P* value |
| --- | --- | --- | --- | --- | --- |
|  | <0.080 | 0.080-0.083 | 0.084-0.086 | >0.086 |  |
| Participants, n | 1904 | 2047 | 2075 | 2353 |  |
| Age, years |  |  |  |  | <0.001 |
| 20-39 | 602(35.68) | 431(23.11) | 232(14.14) | 102(6.15) |  |
| 40-59 | 807(45.73) | 881(49.51) | 708(42.84) | 513(30.32) |  |
| ≥60 | 495(18.59) | 735(27.38) | 1135(43.02) | 1738(63.54) |  |
| Sex, % |  |  |  |  | <0.001 |
| Female | 1257(63.44) | 1025(47.96) | 935(43.11) | 1035(44.30) |  |
| Male | 647(36.56) | 1022(52.04) | 1140(56.89) | 1318(55.70) |  |
| Race/ethnicity, % |  |  |  |  | <0.001 |
| Mexican American | 389(9.67) | 433(10.01) | 402(7.53) | 340(4.98) |  |
| Other Hispanic | 177(5.85) | 200(5.92) | 199(5.36) | 188(3.89) |  |
| Non-Hispanic White | 704(63.42) | 842(66.69) | 993(74.10) | 1383(79.18) |  |
| Non-Hispanic Black | 482(14.45) | 389(10.34) | 310(7.38) | 284(6.03) |  |
| Other race | 152(6.61) | 183(7.04) | 171(5.63) | 158(5.90) |  |
| Education level, % |  |  |  |  | 0.010 |
| Below high school | 514(18.48) | 590(18.97) | 652(20.82) | 837(23.70) |  |
| High school | 453(26.12) | 488(27.10) | 546(29.34) | 565(27.32) |  |
| Above high school | 937(55.40) | 969(53.93) | 877(49.84) | 951(48.98) |  |
| Family PIR, % |  |  |  |  | < 0.001 |
| ≤1.0 | 370(14.01) | 415(13.90) | 458(15.03) | 523(14.75) |  |
| 1.1–3.0 | 839(38.46) | 884(36.76) | 902(38.20) | 1139(45.23) |  |
| >3.0 | 695(47.54) | 748(49.34) | 715(46.77) | 691(40.01) |  |
| Smoking status, % |  |  |  |  | <0.001 |
| Never smoker | 1143(60.13) | 1075(50.79) | 978(43.57) | 965(39.34) |  |
| Former smoker | 432(22.41) | 569(29.64) | 687(32.97) | 898(38.65) |  |
| Current smoker | 329(17.46) | 403(19.58) | 410(23.46) | 490(22.01) |  |
| Drinking status, % |  |  |  |  | <0.001 |
| Nondrinker | 501(21.95) | 493(19.28) | 554(22.05) | 681(25.95) |  |
| Low-to-moderate drinker | 1300(72.67) | 1400(71.33) | 1372(69.34) | 1474(63.43) |  |
| Heavy drinker | 103(5.38) | 154(9.39) | 149(8.61) | 198(10.62) |  |
| Physical activity, % |  |  |  |  | <0.001 |
| Inactive | 532(24.23) | 554(22.62) | 700(28.11) | 921(34.54) |  |
| Insufficiently active | 766(42.77) | 779(41.11) | 744(40.49) | 792(36.27) |  |
| Active | 606(33.00) | 714(36.27) | 631(31.40) | 640(29.19) |  |
| Healthy eating index | 48.53(39.29,58.04) | 48.62(39.58,57.66) | 48.72(39.44,59.06) | 49.57(40.64,58.83) | 0.150 |
| Charlson comorbidity index | 0.94(0.04) | 1.18(0.04) | 1.47(0.04) | 2.03(0.06) | <0.001 |
| Waist circumference, cm | 102.5(94.9,112.0) | 105.6(97.8,115.3) | 107.6(99.6,117.7) | 110.7(101.7,121.2) | <0.001 |
| Weight, kg | 92.4(80.6,106.7) | 90.5(78.7,105.2) | 89.4(77.5,102.6) | 85.9(72.9,100.3) | <0.001 |
| Height, cm | 165.8(159.6,173.3) | 169.3(161.5,176.8) | 170.6(161.7,177.8) | 169.4(161.3,177.1) | <0.001 |
| Body mass index, kg/m^2^ | 32.60(29.10,37.85) | 31.10(27.90,35.80) | 30.54(27.30,34.80) | 29.60(26.20,33.97) | <0.001 |
| HDL-C, mg/dL | 1.11(0.93,1.29) | 1.11(0.96,1.32) | 1.14(0.96,1.34) | 1.16(0.98,1.39) | 0.020 |
| Triglyceride, mg/dL | 162.0(110.0,215.0) | 163.0(112.0,225.0) | 155.0(108.0,217.0) | 155.0(106.0,213.0) | 0.050 |
| Fasting plasma glucose, mg/dL | 105.0(99.0,113.0) | 107.0(101.0,118.0) | 108.2(102.0,122.0) | 112.0(103.0,130.0) | <0.001 |
| Anthropometric indexes |  |  |  |  |  |
| ABSI | 0.08(0.08,0.08) | 0.08(0.08,0.08) | 0.08(0.08,0.09) | 0.09(0.09,0.09) | <0.001 |
| BRI | 5.67(4.64,7.18) | 5.89(4.83,7.52) | 6.20(5.12,7.69) | 6.73(5.50,8.23) | <0.001 |
| CMI | 0.22(0.16,0.30) | 0.23(0.16,0.31) | 0.22(0.16,0.31) | 0.23(0.17,0.32) | 0.010 |
| VAI | 2.68(1.72,3.90) | 2.67(1.80,3.97) | 2.64(1.65,4.03) | 2.58(1.65,4.18) | 0.610 |
| WTI | 9.03(8.64,9.35) | 9.07(8.71,9.40) | 9.05(8.68,9.39) | 9.05(8.68,9.40) | 0.130 |
| LAP | 76.05(48.94,111.36) | 80.71(53.87,119.06) | 81.72(52.54,119.14) | 82.77(55.44,125.51) | < 0.001 |
| AIP | 3.74(2.25,5.55) | 3.69(2.45,5.61) | 3.55(2.24,5.35) | 3.44(2.14,5.17) | 0.010 |
| TyG | 9.05(8.70,9.36) | 9.08(8.75,9.48) | 9.09(8.71,9.43) | 9.09(8.73,9.45) | 0.040 |
| All-cause mortality, % |  |  |  |  | <0.001 |
| No | 1726(92.49) | 1772(88.99) | 1629(82.23) | 1554(73.12) |  |
| Yes | 178(7.51) | 275(11.01) | 446(17.77) | 799(26.88) |  |
| CCD mortality, % |  |  |  |  | <0.001 |
| No | 1845(97.78) | 1950(96.22) | 1929(94.14) | 2087(91.73) |  |
| Yes | 59(2.22) | 97(3.78) | 146(5.86) | 266(8.27) |  |
| Follow-up time, years | 10.33(5.75,15.33) | 8.92(4.50,13.75) | 8.25(4.25,13.08) | 6.75(3.42,11.25) | <0.001 |

Abbreviations: PIR, poverty income ratio; HDL-C, high-density lipoprotein cholesterol. ABSI, a body shape index; BRI, body roundness index; CMI, cardiometabolic index; VAI, visceral adiposity index; WTI, waist triglyceride index; LAP, lipid accumulation product; AIP, atherogenic index of plasma; TyG, triglyceride-glucose index; CCD, cardio-cerebrovascular disease.

Normally distributed continuous variables are described as means ± SEs, and continuous variables without a normal distribution are presented as medians [interquartile ranges]. Sampling weights were applied for calculation of demographic descriptive statistics; N reflect the study sample while percentages reflect the survey-weighted data.

**Table S3.** HR (95% CIs) of all-cause mortality according to quartiles of anthropometric indexes among individuals with metabolic syndrome in NHANES 1999–2018.

|  | Quartiles of anthropometric indexes | | | | *P*_trend_ |
| --- | --- | --- | --- | --- | --- |
|  | Quartile 1 | Quartile 2 | Quartile 3 | Quartile 4 |  |
| **BRI** | |  |  |  |  |
| Crude | 1 [Reference] | 1.070 (0.900-1.272) | 1.219 (1.021-1.454) | 1.330 (1.135-1.559) | <0.001 |
| Model 1 | 1 [Reference] | 0.924 (0.781-1.094) | 0.969 (0.791-1.187) | 1.204 (1.027-1.411) | 0.011 |
| Model 2 | 1 [Reference] | 0.863 (0.727-1.025) | 0.878 (0.722-1.069) | 0.980 (0.837-1.147) | 0.846 |
| **CMI** | |  |  |  |  |
| Crude | 1 [Reference] | 0.902 (0.755-1.076) | 0.783 (0.656-0.935) | 0.859 (0.739-0.998) | 0.077 |
| Model 1 | 1 [Reference] | 0.982 (0.826-1.167) | 0.910 (0.765-1.084) | 1.107 (0.950-1.290) | 0.144 |
| Model 2 | 1 [Reference] | 0.902 (0.759-1.072) | 0.758 (0.641-0.896) | 0.909 (0.795-1.040) | 0.301 |
| **VAI** |  |  |  |  |  |
| Crude | 1 [Reference] | 0.798 (0.682-0.934) | 0.626 (0.526-0.746) | 0.648 (0.561-0.747) | <0.001 |
| Model 1 | 1 [Reference] | 0.966 (0.826-1.130) | 0.900 (0.751-1.078) | 1.090 (0.938-1.266) | 0.165 |
| Model 2 | 1 [Reference] | 0.892 (0.763-1.043) | 0.784 (0.662-0.929) | 0.929 (0.806-1.071) | 0.635 |
| **WTI** |  |  |  |  |  |
| Crude | 1 [Reference] | 0.764 (0.639-0.913) | 0.720 (0.599-0.865) | 0.656 (0.566-0.761) | <0.001 |
| Model 1 | 1 [Reference] | 0.833 (0.706-0.982) | 0.867 (0.730-1.030) | 0.890 (0.770-1.029) | 0.198 |
| Model 2 | 1 [Reference] | 0.763 (0.646-0.900) | 0.741 (0.623-0.882) | 0.790 (0.689-0.906) | 0.002 |
| **LAP** |  |  |  |  |  |
| Crude | 1 [Reference] | 0.827 (0.719-0.951) | 0.629 (0.523-0.757) | 0.698 (0.588-0.828) | <0.001 |
| Model 1 | 1 [Reference] | 0.867 (0.758-0.991) | 0.733 (0.616-0.873) | 0.926 (0.790-1.085) | 0.640 |
| Model 2 | 1 [Reference] | 0.815 (0.713-0.932) | 0.639 (0.536-0.763) | 0.787 (0.685-0.904) | 0.016 |
| AIP |  |  |  |  |  |
| Crude | 1 [Reference] | 0.802 (0.678-0.949) | 0.613 (0.505-0.743) | 0.606 (0.518-0.708) | <0.001 |
| Model 1 | 1 [Reference] | 0.862 (0.730-1.017) | 0.857 (0.705-1.040) | 0.984 (0.839-1.153) | 0.679 |
| Model 2 | 1 [Reference] | 0.797 (0.677-0.939) | 0.723 (0.603-0.868) | 0.857 (0.737-0.996) | 0.274 |
| **TyG** |  |  |  |  |  |
| Crude | 1 [Reference] | 0.811 (0.667-0.986) | 0.825 (0.695-0.979) | 0.896 (0.755-1.063) | 0.343 |
| Model 1 | 1 [Reference] | 0.921 (0.760-1.117) | 0.953 (0.800-1.136) | 1.103 (0.929-1.310) | 0.186 |
| Model 2 | 1 [Reference] | 0.873 (0.715-1.065) | 0.858 (0.723-1.018) | 0.890 (0.751-1.056) | 0.212 |

Abbreviations: HR, hazard ratio; CI, confidence interval; BRI, body roundness index; CMI, cardiometabolic index; VAI, visceral adiposity index; WTI, waist triglyceride index; LAP, lipid accumulation product; AIP, atherogenic index of plasma; TyG, triglyceride-glucose index.

Data are presented as HR (95% CI) unless indicated otherwise; Model 1 was adjusted for age (20-39, 40-59, or ≥60), sex (male or female), and race/ethnicity (Mexican American, other Hispanic, non-Hispanic White, non-Hispanic Black, or other race); Model 2 was adjusted as model 1 plus education level (below high school, high school, or above high school), family PIR (≤1.0, 1.1–3.0, or >3.0), smoking status (never smoker, former smoker, or current smoker), drinking status (nondrinker, low-to-moderate drinker, or heavy drinker), physical activity (inactive, insufficiently active, or active), HEI (in quartiles), and CCI (continous).


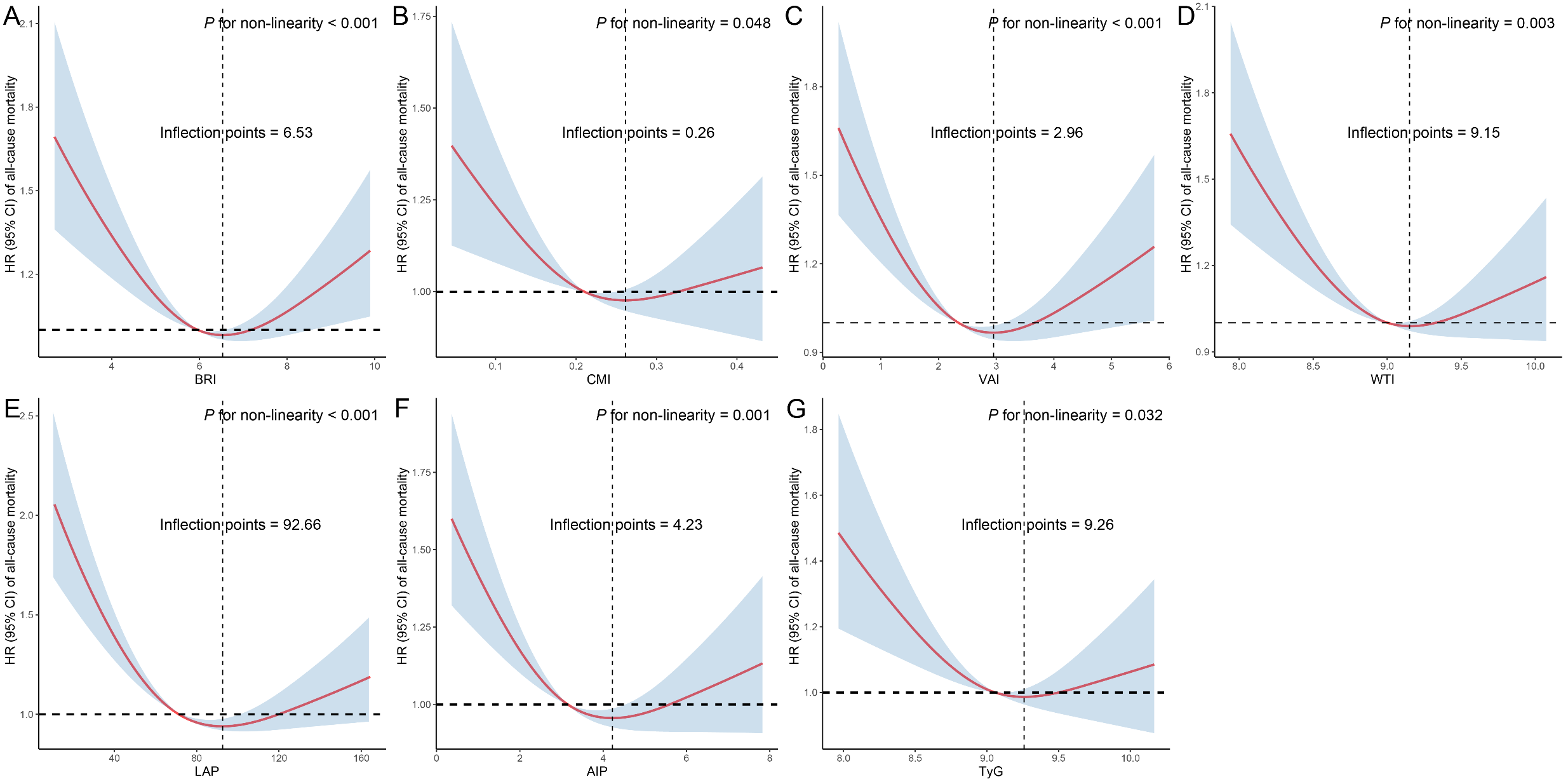


**Figure S2.** Restricted cubic spline (RCS) analysis with multivariate-adjusted associations of anthropometric indexes (A: BRI; B: CMI; C: VAI; D: WTI; E: LAP; F: AIP; and G: TyG) with all-cause mortality among individuals with metabolic syndrome in NHANES 1999–2018. Models are adjusted for age (20-39, 40-59, or ≥60), sex (male or female), race/ethnicity (Mexican American, other Hispanic, non-Hispanic White, non-Hispanic Black, or other race), education level (below high school, high school, or above high school), family PIR (≤1.0, 1.1–3.0, or >3.0), smoking status (never smoker, former smoker, or current smoker), drinking status (nondrinker, low-to-moderate drinker, or heavy drinker), physical activity (inactive, insufficiently active, or active), HEI (in quartiles), and CCI (continous).

Abbreviations: BRI, body roundness index; CMI, cardiometabolic index; VAI, visceral adiposity index; WTI, waist triglyceride index; LAP, lipid accumulation product; AIP, atherogenic index of plasma; TyG, triglyceride-glucose index.

**Table S4.** HR (95% CIs) of cardio-cerebrovascular disease mortality according to quartiles of anthropometric indexes among individuals with metabolic syndrome in NHANES 1999–2018.

|  | Quartiles of anthropometric indexes | | | | *P*_trend_ |
| --- | --- | --- | --- | --- | --- |
|  | Quartile 1 | Quartile 2 | Quartile 3 | Quartile 4 |  |
| **BRI** | |  |  |  |  |
| Crude | 1 [Reference] | 0.998 (0.767-1.298) | 1.174 (0.864-1.593) | 1.398 (1.085-1.800) | 0.005 |
| Model 1 | 1 [Reference] | 0.843 (0.650-1.093) | 0.915 (0.666-1.258) | 1.260 (0.971-1.636) | 0.041 |
| Model 2 | 1 [Reference] | 0.776 (0.603-0.999) | 0.807 (0.584-1.115) | 1.003 (0.777-1.295) | 0.610 |
| **CMI** | |  |  |  |  |
| Crude | 1 [Reference] | 0.867 (0.655-1.146) | 0.778 (0.567-1.068) | 0.817 (0.635-1.051) | 0.175 |
| Model 1 | 1 [Reference] | 0.966 (0.727-1.284) | 0.940 (0.686-1.289) | 1.106 (0.842-1.452) | 0.411 |
| Model 2 | 1 [Reference] | 0.877 (0.658-1.168) | 0.773 (0.567-1.054) | 0.886 (0.676-1.162) | 0.527 |
| **VAI** |  |  |  |  |  |
| Crude | 1 [Reference] | 0.709 (0.522-0.962) | 0.463 (0.331-0.649) | 0.558 (0.431-0.721) | <0.001 |
| Model 1 | 1 [Reference] | 0.891 (0.656-1.210) | 0.709 (0.506-0.993) | 1.028 (0.777-1.359) | 0.692 |
| Model 2 | 1 [Reference] | 0.819 (0.602-1.113) | 0.603 (0.442-0.822) | 0.858 (0.654-1.127) | 0.494 |
| **WTI** |  |  |  |  |  |
| Crude | 1 [Reference] | 0.652 (0.488-0.870) | 0.588 (0.423-0.816) | 0.598 (0.455-0.785) | <0.001 |
| Model 1 | 1 [Reference] | 0.727 (0.552-0.958) | 0.744 (0.542-1.022) | 0.855 (0.641-1.141) | 0.368 |
| Model 2 | 1 [Reference] | 0.669 (0.502-0.892) | 0.621 (0.455-0.847) | 0.757 (0.560-1.024) | 0.098 |
| **LAP** |  |  |  |  |  |
| Crude | 1 [Reference] | 0.646 (0.485-0.860) | 0.515 (0.377-0.705) | 0.638 (0.489-0.833) | 0.019 |
| Model 1 | 1 [Reference] | 0.688 (0.518-0.915) | 0.621 (0.458-0.842) | 0.890 (0.678-1.169) | 0.966 |
| Model 2 | 1 [Reference] | 0.648 (0.483-0.868) | 0.532 (0.395-0.717) | 0.753 (0.571-0.993) | 0.301 |
| AIP |  |  |  |  |  |
| Crude | 1 [Reference] | 0.708 (0.521-0.961) | 0.559 (0.399-0.782) | 0.524 (0.393-0.697) | <0.001 |
| Model 1 | 1 [Reference] | 0.778 (0.568-1.065) | 0.817 (0.587-1.138) | 0.889 (0.645-1.225) | 0.770 |
| Model 2 | 1 [Reference] | 0.723 (0.530-0.986) | 0.674 (0.496-0.916) | 0.762 (0.556-1.044) | 0.256 |
| **TyG** |  |  |  |  |  |
| Crude | 1 [Reference] | 0.691 (0.526-0.908) | 0.791 (0.587-1.066) | 0.886 (0.655-1.197) | 0.738 |
| Model 1 | 1 [Reference] | 0.819 (0.617-1.088) | 0.959 (0.715-1.285) | 1.152 (0.838-1.583) | 0.250 |
| Model 2 | 1 [Reference] | 0.780 (0.580-1.048) | 0.859 (0.634-1.164) | 0.921 (0.677-1.252) | 0.806 |

Abbreviations: HR, hazard ratio; CI, confidence interval; BRI, body roundness index; CMI, cardiometabolic index; VAI, visceral adiposity index; WTI, waist triglyceride index; LAP, lipid accumulation product; AIP, atherogenic index of plasma; TyG, triglyceride-glucose index.

Data are presented as HR (95% CI) unless indicated otherwise; Model 1 was adjusted for age (20-39, 40-59, or ≥60), sex (male or female), and race/ethnicity (Mexican American, other Hispanic, non-Hispanic White, non-Hispanic Black, or other race); Model 2 was adjusted as model 1 plus education level (below high school, high school, or above high school), family PIR (≤1.0, 1.1–3.0, or >3.0), smoking status (never smoker, former smoker, or current smoker), drinking status (nondrinker, low-to-moderate drinker, or heavy drinker), physical activity (inactive, insufficiently active, or active), HEI (in quartiles), and CCI (continous).


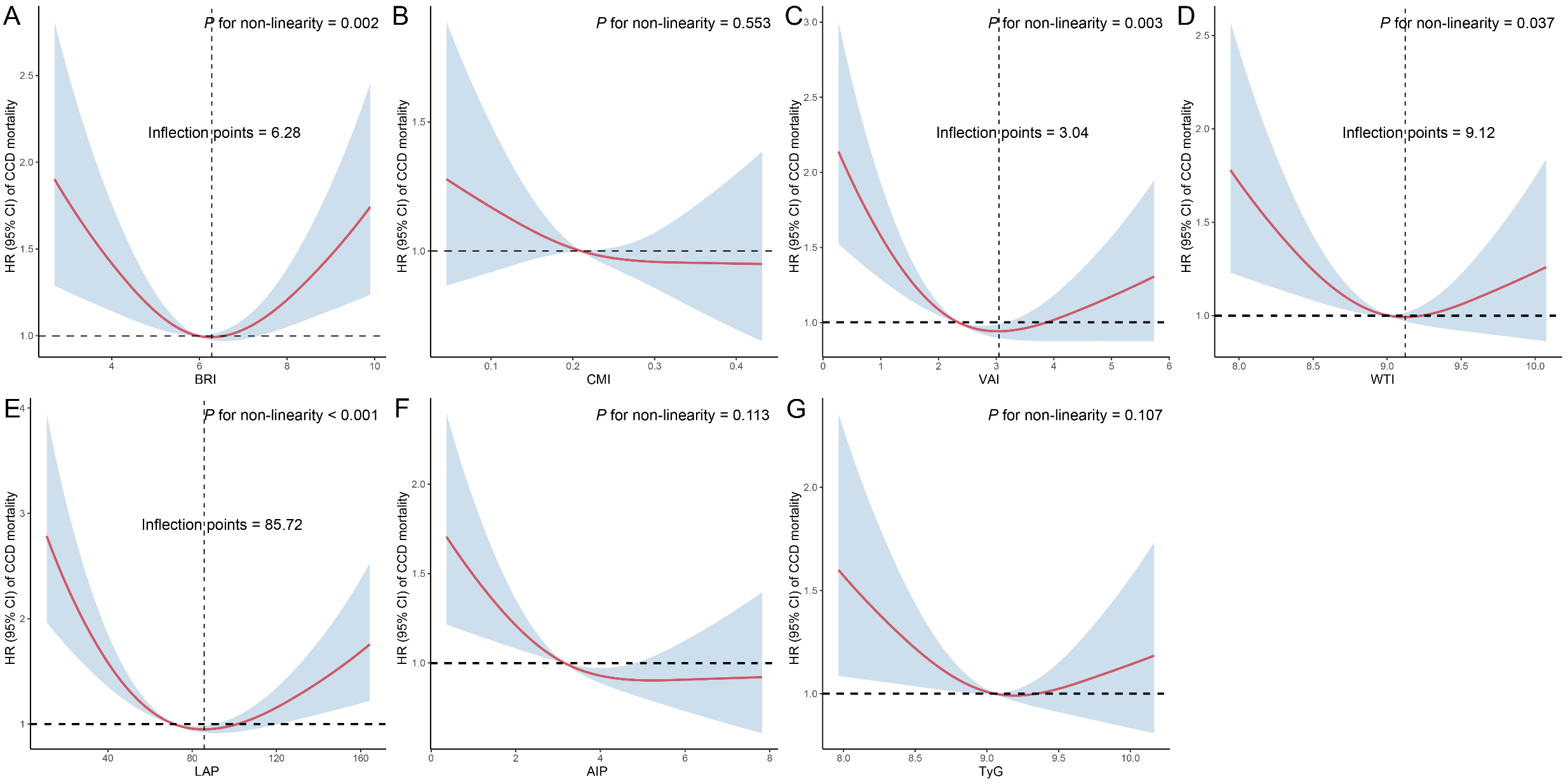


**Figure S3.** Restricted cubic spline (RCS) analysis with multivariate-adjusted associations of anthropometric indexes (A: BRI; B: CMI; C: VAI; D: WTI; E: LAP; F: AIP; and G: TyG) with cardio-cerebrovascular disease mortality among individuals with metabolic syndrome in NHANES 1999–2018. Models are adjusted for age (20-39, 40-59, or ≥60), sex (male or female), race/ethnicity (Mexican American, other Hispanic, non-Hispanic White, non-Hispanic Black, or other race), education level (below high school, high school, or above high school), family PIR (≤1.0, 1.1–3.0, or >3.0), smoking status (never smoker, former smoker, or current smoker), drinking status (nondrinker, low-to-moderate drinker, or heavy drinker), physical activity (inactive, insufficiently active, or active), HEI (in quartiles), and CCI (continous).

Abbreviations: BRI, body roundness index; CMI, cardiometabolic index; VAI, visceral adiposity index; WTI, waist triglyceride index; LAP, lipid accumulation product; AIP, atherogenic index of plasma; TyG, triglyceride-glucose index.

**
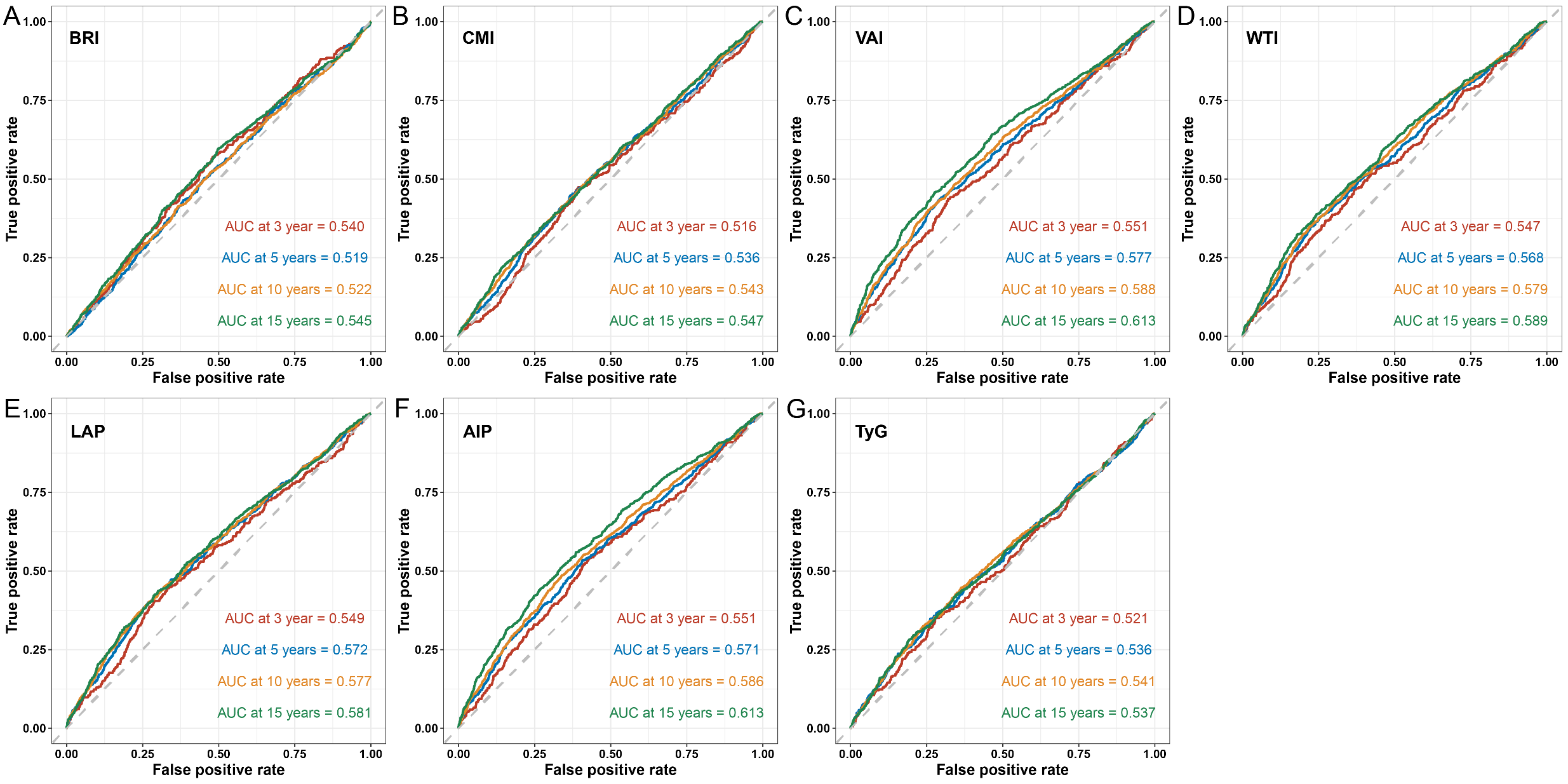
**

**Figure S4.** Predictive value of time-dependent ROC assessment of anthropometric indexes (A: BRI; B: CMI; C: VAI; D: WTI; E: LAP; F: AIP; and G: TyG) for 3-, 5-, 10-, and 15-year all-cause mortality. Abbreviations: BRI, body roundness index; CMI, cardiometabolic index; VAI, visceral adiposity index; WTI, waist triglyceride index; LAP, lipid accumulation product; AIP, atherogenic index of plasma; TyG, triglyceride-glucose index.

**
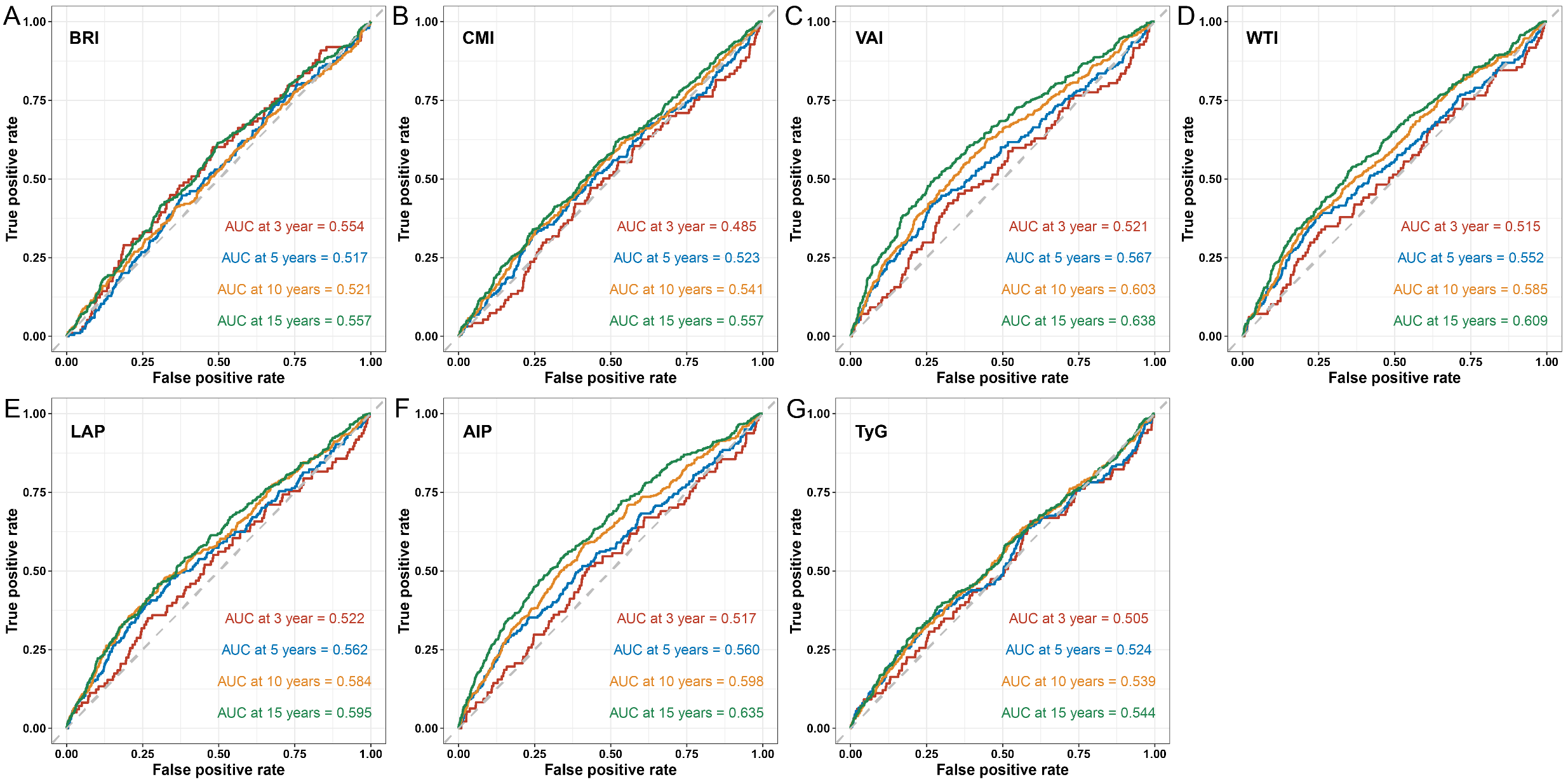
**

**Figure S5.** Predictive value of time-dependent ROC assessment of anthropometric indexes (A: BRI; B: CMI; C: VAI; D: WTI; E: LAP; F: AIP; and G: TyG) for 3-, 5-, 10-, and 15-year cardio-cerebrovascular disease mortality. Abbreviations: BRI, body roundness index; CMI, cardiometabolic index; VAI, visceral adiposity index; WTI, waist triglyceride index; LAP, lipid accumulation product; AIP, atherogenic index of plasma; TyG, triglyceride-glucose index.

**Table S5.** Improvement of risk prediction by adding ABSI to fully adjusted model.

|  | 3 years AUC (95% CI) | 5 years AUC (95% CI) | 10 years AUC (95% CI) | 15 years AUC (95% CI) |
| --- | --- | --- | --- | --- |
| **All-cause Mortality** | |  |  |  |
| Model* | 80.39 (78.28-82.51) | 79.58 (77.86-81.31) | 80.89 (79.50-82.29) | 83.42 (81.85-84.96) |
| Model + ABSI | 81.73 (79.67-83.81) | 80.83 (79.12-82.54) | 81.89 (80.55-83.25) | 84.39 (82.91-85.88) |
| *P* value | <0.001 | <0.001 | <0.001 | <0.001 |
| **CCD Mortality** | |  |  |  |
| Model* | 79.99 (76.16-83.82) | 81.96 (79.42-84.49) | 82.13 (80.09-84.15) | 84.38 (82.08-86.69) |
| Model + ABSI | 81.66 (77.93-85.39) | 83.48 (81.03-85.94) | 83.20 (81.22-85.21) | 85.16 (82.89-87.45) |
| *P* value | <0.001 | <0.001 | <0.001 | <0.001 |

Abbreviations: CI, confidence interval; AUC, area under the curve; ABSI, a body shape index.

* Model was adjusted for age (20-39, 40-59, or ≥60), sex (male or female), race/ethnicity (Mexican American, other Hispanic, non-Hispanic White, non-Hispanic Black, or other race), education level (below high school, high school, or above high school), family PIR (≤1.0, 1.1–3.0, or >3.0), smoking status (never smoker, former smoker, or current smoker), drinking status (nondrinker, low-to-moderate drinker, or heavy drinker), physical activity (inactive, insufficiently active, or active), HEI (in quartiles), and CCI (continous).

**Table S6.** HR (95% CIs) of mortality according to quartiles of ABSI after excluding participants who died within one years of follow-up among individuals with metabolic syndrome in NHANES 1999–2018 (n=8,294).

|  | Quartiles of ABSI levels | | | | *P*_trend_ |
| --- | --- | --- | --- | --- | --- |
|  | <0.080 | 0.080-0.083 | 0.084-0.086 | >0.086 |  |
| All-cause Mortality | |  |  |  |  |
| Crude | 1 [Reference] | 1.697 (1.325-2.173) | 2.766 (2.209-3.463) | 5.179 (4.175-6.425) | <0.001 |
| Model 1 | 1 [Reference] | 1.327 (1.035-1.702) | 1.659 (1.327-2.073) | 2.307 (1.838-2.895) | <0.001 |
| Model 2 | 1 [Reference] | 1.230 (0.953-1.588) | 1.476 (1.181-1.845) | 1.707 (1.357-2.148) | <0.001 |
| Cardio-cerebrovascular Disease Mortality | | |  |  |  |
| Crude | 1 [Reference] | 2.015 (1.418-2.862) | 3.032 (2.145-4.287) | 5.290 (3.957-7.073) | <0.001 |
| Model 1 | 1 [Reference] | 1.518 (1.083-2.128) | 1.678 (1.191-2.365) | 2.142 (1.562-2.939) | <0.001 |
| Model 2 | 1 [Reference] | 1.443 (1.026-2.029) | 1.526 (1.084-2.148) | 1.615 (1.174-2.221) | 0.021 |

Abbreviations: HR, hazard ratio; CI, confidence interval; ABSI, a body shape index.

Model 1 was adjusted for age (20-39, 40-59, or ≥60), sex (male or female), and race/ethnicity (Mexican American, other Hispanic, non-Hispanic White, non-Hispanic Black, or other race); Model 2 was adjusted as model 1 plus education level (below high school, high school, or above high school), family PIR (≤1.0, 1.1–3.0, or >3.0), smoking status (never smoker, former smoker, or current smoker), drinking status (nondrinker, low-to-moderate drinker, or heavy drinker), physical activity (inactive, insufficiently active, or active), HEI (in quartiles), and CCI (continous).

**Table S7.** HR (95% CIs) of mortality according to quartiles of ABSI after excluding participants who had CVD history at baseline among individuals with metabolic syndrome in NHANES 1999–2018 (n=6,109).

|  | Quartiles of ABSI levels | | | | *P*_trend_ |
| --- | --- | --- | --- | --- | --- |
|  | <0.080 | 0.080-0.083 | 0.084-0.086 | >0.086 |  |
| All-cause Mortality | |  |  |  |  |
| Crude | 1 [Reference] | 1.482 (1.113-1.973) | 2.556 (1.938-3.372) | 4.626 (3.571-5.992) | <0.001 |
| Model 1 | 1 [Reference] | 1.174 (0.873-1.580) | 1.569 (1.186-2.076) | 2.103 (1.583-2.795) | <0.001 |
| Model 2 | 1 [Reference] | 1.072 (0.795-1.446) | 1.365 (1.029-1.810) | 1.548 (1.154-2.078) | <0.001 |
| Cardio-cerebrovascular Disease Mortality | | |  |  |  |
| Crude | 1 [Reference] | 1.396 (0.845-2.306) | 2.708 (1.611-4.553) | 5.093 (3.427-7.570) | <0.001 |
| Model 1 | 1 [Reference] | 1.062 (0.655-1.722) | 1.501 (0.891-2.528) | 2.033 (1.303-3.172) | <0.001 |
| Model 2 | 1 [Reference] | 0.992 (0.616-1.599) | 1.358 (0.802-2.298) | 1.591 (1.000-2.530) | 0.022 |

Abbreviations: HR, hazard ratio; CI, confidence interval; ABSI, a body shape index.

Model 1 was adjusted for age (20-39, 40-59, or ≥60), sex (male or female), and race/ethnicity (Mexican American, other Hispanic, non-Hispanic White, non-Hispanic Black, or other race); Model 2 was adjusted as model 1 plus education level (below high school, high school, or above high school), family PIR (≤1.0, 1.1–3.0, or >3.0), smoking status (never smoker, former smoker, or current smoker), drinking status (nondrinker, low-to-moderate drinker, or heavy drinker), physical activity (inactive, insufficiently active, or active), HEI (in quartiles), and CCI (continous).

**Table S8.** HR (95% CIs) of mortality according to quartiles of ABSI after excluding participants who had cancer history at baseline among individuals with metabolic syndrome in NHANES 1999–2018 (n=7,341).

|  | Quartiles of ABSI levels | | | | *P*_trend_ |
| --- | --- | --- | --- | --- | --- |
|  | <0.080 | 0.080-0.083 | 0.084-0.086 | >0.086 |  |
| All-cause Mortality | |  |  |  |  |
| Crude | 1 [Reference] | 1.665 (1.284-2.157) | 2.791 (2.173-3.584) | 5.158 (4.098-6.493) | <0.001 |
| Model 1 | 1 [Reference] | 1.334 (1.040-1.711) | 1.752 (1.376-2.230) | 2.407 (1.900-3.050) | <0.001 |
| Model 2 | 1 [Reference] | 1.239 (0.966-1.588) | 1.533 (1.210-1.944) | 1.760 (1.377-2.249) | <0.001 |
| Cardio-cerebrovascular Disease Mortality | | |  |  |  |
| Crude | 1 [Reference] | 2.006 (1.355-2.969) | 3.283 (2.224-4.846) | 5.170 (3.763-7.103) | <0.001 |
| Model 1 | 1 [Reference] | 1.519 (1.036-2.227) | 1.829 (1.242-2.691) | 2.092 (1.487-2.944) | <0.001 |
| Model 2 | 1 [Reference] | 1.451 (0.995-2.115) | 1.669 (1.130-2.464) | 1.568 (1.098-2.240) | 0.044 |

Abbreviations: HR, hazard ratio; CI, confidence interval; ABSI, a body shape index.

Model 1 was adjusted for age (20-39, 40-59, or ≥60), sex (male or female), and race/ethnicity (Mexican American, other Hispanic, non-Hispanic White, non-Hispanic Black, or other race); Model 2 was adjusted as model 1 plus education level (below high school, high school, or above high school), family PIR (≤1.0, 1.1–3.0, or >3.0), smoking status (never smoker, former smoker, or current smoker), drinking status (nondrinker, low-to-moderate drinker, or heavy drinker), physical activity (inactive, insufficiently active, or active), HEI (in quartiles), and CCI (continous).
